# Advances in Understanding TKS4 and TKS5: Molecular Scaffolds Regulating Cellular Processes from Podosome and Invadopodium Formation to Differentiation and Tissue Homeostasis

**DOI:** 10.3390/ijms21218117

**Published:** 2020-10-30

**Authors:** Gyöngyi Kudlik, Tamás Takács, László Radnai, Anita Kurilla, Bálint Szeder, Kitti Koprivanacz, Balázs L. Merő, László Buday, Virag Vas

**Affiliations:** 1 Institute of Enzymology, Research Centre for Natural Sciences, 1117 Budapest, Hungary; kudlik.gyongyi@ttk.hu (G.K.); takacs.tamas@ttk.hu (T.T.); lradnai@scripps.edu (L.R.); kurilla.anita@abc.naik.hu (A.K.); szeder.balint@ttk.mta.hu (B.S.); koprivanacz.kitti@ttk.hu (K.K.); mero.balazs@ttk.mta.hu (B.L.M.); buday.laszlo@ttk.mta.hu (L.B.); 2 Department of Molecular Medicine, The Scripps Research Institute, Jupiter, FL 33458, USA; 3 Department of Neuroscience, The Scripps Research Institute, Jupiter, FL 33458, USA; 4 Department of Medical Chemistry, Semmelweis University Medical School, 1085 Budapest, Hungary

**Keywords:** scaffold protein, tyrosine kinase substrates, TKS4, TKS5, invasion, mesenchymal stem cells, adipose tissue, bone homeostasis, epithelial–mesenchymal transition

## Abstract

Scaffold proteins are typically thought of as multi-domain “bridging molecules.” They serve as crucial regulators of key signaling events by simultaneously binding multiple participants involved in specific signaling pathways. In the case of epidermal growth factor (EGF)-epidermal growth factor receptor (EGFR) binding, the activated EGFR contacts cytosolic SRC tyrosine-kinase, which then becomes activated. This process leads to the phosphorylation of SRC-substrates, including the tyrosine kinase substrates (TKS) scaffold proteins. The TKS proteins serve as a platform for the recruitment of key players in EGFR signal transduction, promoting cell spreading and migration. The TKS4 and the TKS5 scaffold proteins are tyrosine kinase substrates with four or five SH3 domains, respectively. Their structural features allow them to recruit and bind a variety of signaling proteins and to anchor them to the cytoplasmic surface of the cell membrane. Until recently, TKS4 and TKS5 had been recognized for their involvement in cellular motility, reactive oxygen species-dependent processes, and embryonic development, among others. However, a number of novel functions have been discovered for these molecules in recent years. In this review, we attempt to cover the diverse nature of the TKS molecules by discussing their structure, regulation by SRC kinase, relevant signaling pathways, and interaction partners, as well as their involvement in cellular processes, including migration, invasion, differentiation, and adipose tissue and bone homeostasis. We also describe related pathologies and the established mouse models.

## 1. Introduction

**Figure 1:**
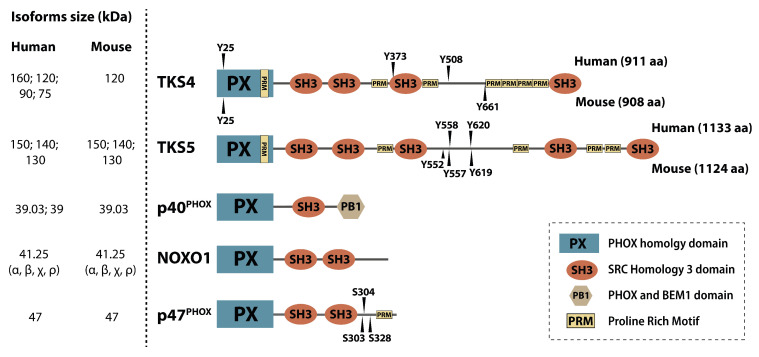
Members of the p47 organizer protein family. The p47 organizer family consists of five structurally similar adaptor/scaffold proteins containing an N-terminal PX domain followed by several SH3 domains, namely p40^phox^, NOXO1, p47^phox^, TKS4, and TKS5. Experimentally confirmed SRC kinase tyrosine phosphorylation sites (“Y”) in the human and mouse TKS proteins are shown above and below the depicted domain architecture, respectively.

Scaffold proteins modulate intracellular signaling by bringing regulatory proteins, enzymes, or cytoskeletal structures in close proximity [1]. TKS molecules are large scaffold proteins earning their name from the early observation that they serve as tyrosine kinase substrates of SRC kinase [2,3,4]. TKS4 and TKS5 contain one Phox Homology (PX) domain, conserved linear motifs, e.g., several proline-rich motifs (PRMs), and four or five SRC Homology 3 (SH3) domains, respectively. Other names for TKS5 are SH3 and PX domain-containing protein 2A (SH3PXD2A) and Five SH3 domains (FISH), while TKS4 is also known as SH3 and PX domain-containing protein 2B (SH3PXD2B), Homolog of FISH (HOFI), and a factor of adipocyte differentiation 49 (Fad49), reflecting some of their known characteristics [3,5]. The main function of the PX domain is to link the TKS scaffold proteins to the cell membrane via phosphoinositide binding [2,6]. The SH3 domains serve as docking sites for signaling molecules and mediate protein-protein interactions [7]. It is likely that the PRMs of the TKS proteins represent contact sites for SH3 domain-containing molecules (Figure 1). The TKS proteins are phylogenetically related and are expressed in vertebrates, and TKS-like genes are widely present in invertebrates [8]. TKS scaffold proteins are broadly expressed in tissues except for the testis for TKS4, and the spleen and testis for TKS5 [2,3]. They are also expressed in several transformed cell lines [2,9].

In this review, we summarize the current knowledge of the properties of TKS4 and TKS5 and their involvement in specific cellular processes, including growth factor signaling, formation of actin-rich membrane protrusions, and generation of reactive oxygen species by regulating NADPH oxidase (NOX) transmembrane enzyme complexes. We also provide an overview of the function of TKS proteins in cancer metastasis and genetic diseases.

## 2. The Regulated Localization of TKS Proteins Determines Their Signal Recruiting Function

Both TKS4 and TKS5 have a cytoplasmic, inactive state, and a membrane-bound, active state in cells. The transition between these states is most likely regulated by phosphorylation [2,10,11]. Direct evidence for these conformational states is still limited [12]. However, this hypothesis is supported by the auto-inhibitory intramolecular interactions known to regulate p47^phox^, a homologous protein that is structurally highly similar to the N-terminal regions of TKS4 and TKS5. Based on the similarities in their domain architecture, all of these proteins belong to the p47-related organizer protein family [13] (Figure 1). In mammals, the p47-related organizer family consists of five members: p40^phox^, NOXO1 and p47^phox^, TKS4, and TKS5 [1]. These proteins share many functional and conformational similarities [4,14]. A common feature is the presence of an N-terminal PX domain, followed by one or more SH3 domains (Figure 1). An intramolecular regulatory mechanism was first described for p47^phox^. In the auto-inhibited state, its first and second SH3 domains (“tandem SH3”) bind a specific proline-rich motif within the C-terminal region. The assembly of this auto-inhibitory organization makes the PX domain inaccessible for phosphatidylinositol phosphates. Therefore, p47^phox^ remains in the cytoplasm [15,16,17]. The autoinhibitory interaction is disrupted by phosphorylation of several C-terminal serine residues located in close proximity to the proline-rich motif. Consequently, the tandem SH3 domains release the proline-rich motif and become available to bind an interacting partner, p22^phox^. In association with these events, the locked PX domain is released and anchors the protein to the membrane via phospholipid binding [18].

The intramolecular elements known to be necessary for the autoinhibited state of p47^phox^ (the tandem SH3 domains and the proline-rich autoinhibitory region) are conserved and are present in both TKS4 and TKS5 [14]. Therefore, the similarities between the structures of the p47^phox^ and TKS proteins support the idea that similar intramolecular interactions could regulate their functional states. A study by Abram and colleagues supports the existence of intramolecular regulation in TKS5 [6]. Based on their results, they speculated that, when TKS5 is in its auto-inhibited conformation, its PX lipid recognition module is masked, and the molecule is distributed diffusely in the cell. Upon SRC phosphorylation on tyrosine residues, the PX domain is released, thus becoming available to bind to membrane lipids, resulting in translocation of TKS5 to the cell membrane [6]. Simultaneously, the SH3 domains can bind to signaling proteins to recruit them to the cell membrane, allowing intracellular signal transduction [6,12,19].

## 3. TKS4 and TKS5 Affect Multiple Biological Processes from Growth Factor Receptor Signaling to Metastasis to Tissue Homeostasis

### 3.1. EGFR Signaling via TKS4 and TKS5

Receptor tyrosine kinases (RTK) are transmembrane proteins that control several cellular processes, ranging from proliferation to differentiation and cell migration. Following the binding of their extracellular ligands, RTKs dimerize, undergo auto-phosphorylation on multiple tyrosine residues in their cytoplasmic region, and associate with intracellular signaling molecules. Diverse molecular cascades transmit the signal from RTKs to their final effector molecules, ultimately leading to the modulation of distinct biological processes within the cell [20].

**Figure 2:**
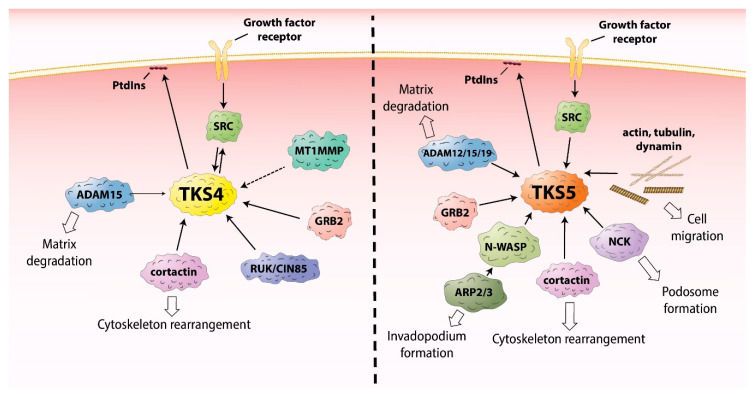
The role of TKS proteins in the recruitment of signaling molecules. ADAM12/15/19–a disintegrin and metalloprotease 12/15/19, ARP2/3–actin-related protein 2/3, GRB2–growth factor receptor binding protein 2, MT1MMP–membrane type 1 matrix metalloprotease, NCK–non-catalytic region of tyrosine kinase adaptor protein, N-WASP–neural Wiskott-Aldrich syndrome protein, PtdIns–phosphatidylinositol, RUK/CIN85–regulator of ubiquitous kinase/Cbl-interacting protein of 85 kDa, SRC–proto-oncogene tyrosine-protein kinase Src.

Epidermal growth factor receptor (EGFR) is one of the most well-studied RTKS. Upon activation, it initiates several signal transduction cascades, including the RAS-RAF-MEK, phosphatidylinositol 3 (PI3)-kinase-AKT, PLCγ, and JAK-STAT pathways [21]. Moreover, active EGFR binds cytosolic SRC tyrosine-kinase, which then becomes activated [22,23,24,25]. This process leads to the phosphorylation of SRC-substrates, including the TKS scaffold proteins, which are known to be involved in EGFR signaling [11,26,27]. The TKS proteins serve as a platform for the recruitment of key players in EGFR signal transduction (Figure 2, Table 1), promoting cell spreading and migration [9,11,28,29,30]. In response to EGFR activation, PI3 kinases are activated, and lipids are phosphorylated in the plasma membrane. For example, phosphatidylinositol (4,5)-bisphosphate (PI(4,5)P_2_) is converted to phosphatidylinositol (3,4,5)-trisphosphate (PI(3,4,5)P_3_) [31]. According to a model proposed by Bögel et al., the phosphorylated lipid residues anchor the PX domain of TKS4 and translocate the scaffold protein from the cytoplasm to the plasma membrane [11]. On the other arm of the signaling pathway, SRC kinase is also activated by binding to the intracellular tail of EGFR [22,23,24,25] subsequently phosphorylating tyrosine residues on TKS4 (i.e., Tyr25, Tyr373, Tyr508) (Figure 1) [2]. Phosphorylated TKS4 can bind activated SRC by interacting with both its SH2 and SH3 domains. In this complex, SRC remains active for a prolonged period of time and may phosphorylate multiple downstream molecules/partners [32]. This direct interaction between TKS4 with SRC was shown to involve the proline-rich region PSRPLPDAP (residues 466–474) and the tyrosine-phosphorylated pYEEI motif (residues 508–511) of TKS4 (both located between the third and fourth SH3 domains) and the SH3 and SH2 domains of SRC, respectively [32]. Upon PI3 kinase activation, TKS5 also translocates to the plasma membrane in epidermal growth factor (EGF)-stimulated cells [26]. The PX domain of both TKS4 and 5 was found to be essential for the participation of the molecules in EGFR signaling and for the phosphorylation of TKS4 and 5 by activated SRC [11,26]. TKS4 forms a complex with EGFR in which either SRC or a yet unidentified protein may serve as a bridge between the two molecules [11,32]. For example, growth factor receptor binding protein 2 (GRB2) has been identified as a binding partner of both EGFR and TKS4 [28]. No strong interaction between TKS5 and EGFR or SRC has been detected so far, suggesting that, despite their structural similarities, there is only a partial overlap between the regulation of TKS4 and TKS5 in EGF signaling [11,26].

In recent years, more possible interaction partners of TKS4 have been identified (Table 1a). One possible partner is cortactin [9], a well-known substrate of SRC localized to cortical actin structures within cells. Cortactin can bind the actin-related protein-2/3 (ARP2/3) and neural Wiskott-Aldrich syndrome protein (N-WASP) proteins, and it mediates actin polymerization [75,76,77]. Therefore, TKS4 was expected to be involved in EGFR signaling-mediated actin cytoskeleton assembly and rearrangement. This proposed mechanism was confirmed by Lányi et al. [9]. They found that, in response to EGF stimulation, TKS4 associates with cellular motility-associated membrane ruffles. They also showed that, when constitutively active SRC is present, TKS4 accumulates in podosomes (actin-rich membrane protrusions involved in cell motility, see below) while forming a complex with SRC and cortactin [9]. TKS5 has also been reported to bind cortactin and other proteins important in the regulation of actin cytoskeleton assembly, including N-WASP, non-catalytic region of tyrosine kinase adaptor protein (NCK), and GRB2 (for a full list, see Table 1b) [10,29,52,78,79].

### 3.2. Molecular Organizers of Podosome and Invadopodium Assembly

Podosomes and invadopodia are dynamically formed actin-rich protrusions formed on the ventral surface of cells facing the extracellular matrix (ECM). Both structures share the function of motility promotion and pericellular proteolytic activity [80]. The migration of normal cells is driven by podosomes, specialized structures that allow cells to adhere to and enter into their surroundings [81]. Podosomes are formed by a variety of different cells under normal circumstances, including endothelial cells, smooth muscle cells, osteoclasts, macrophages, and dendritic cells [82,83]. Invadopodia, by contrast, are used by cancer cells to break ECM barriers and metastasize. Via a coordinated stepwise process at the site of these protruding membrane structures, matrix metalloproteinases accumulate and are secreted into the extracellular space, leading to ECM degradation to facilitate invasion [84].

Both TKS proteins have been implicated in regulating podosome/invadopodium formation and function [2,3,27,85,86]. The processes involved in the assembly of these structures share many similarities. Depending on the cell type, the sequential process of podosome/invadopodium assembly can be initiated by cell adhesion via integrins [87], vascular endothelial growth factor (VEGF) [88], platelet-derived growth factor (PDGF) [89], transforming growth factor beta (TGFβ) [90], keratinocyte growth factor (KGF) [91], colony-stimulating factor-1 (CSF-1) [92], or EGF-derived [93] signals. Upon receptor tyrosine kinase activation, the non-receptor c-SRC kinase becomes active and phosphorylates several substrates, including the TKS proteins, cortactin, N-WASP, focal adhesion kinase (FAK), and other signaling molecules [94,95]. TKS5 has a key role at this point in podosome precursor formation [29]. The PX domain of TKS5 is responsible for docking the molecule to membranes by binding PtdIns(3)P or PtdIns(3,4)P_2_ [6]. During podosome formation, TKS5 is recruited to the plasma membrane by PtdIns(3,4)P_2_ and the GRB2 adaptor while binding to PtdIns(3,4)P_2_ via its PX domain [29]. In this way, the TKS5/GRB2 complex cooperates in the recruitment of proteins necessary for the final podosome-maturation steps. Among the recruited proteins, N-WASP and TKS5 binding has been analyzed in detail and it was experimentally proven that the strong protein interaction is mediated by all five SH3 domains of TKS5, stimulating robust N-WASP accumulation at the adhesion site (Table 1b) [29]. N-WASP is a multi-functional protein containing several different protein subunits that interact with the small GTPase CDC42, PtdIns(4,5)P_2_, and the actin regulatory complex ARP2/3. Upon N-WASP activation and ARP2/3 association, new actin filament polymerization can begin [96,97]. TKS5 functions as a platform to recruit the ARP2/3 complex and ultimately facilitates actin cytoskeleton rearrangement. Finally, the newly formed actin branches allow the cells to change shape and protrude podosomes (Figure 2) [98]. Meanwhile, the other TKS protein TKS4 is phosphorylated by SRC kinase and is recruited to the site of the developing podosome, where it is directly anchored to phosphoinositide residues via its PX domain. The suggested function of TKS4 in podosome maturation is to recruit matrix metalloproteases (MMPs) to the protruding membrane edge and to specifically allow membrane type 1 matrix metalloprotease (MT1-MMP) activation [2]. Buschman et al. showed that loss of TKS4 results in incomplete podosome assembly in which most of the known podosome proteins colocalize at the site of podosome formation but fail to associate with the filamentous actin. Furthermore, MT1-MMP failed to localize to the sites of the pre-podosome structures, resulting in decreased ECM degradation. TKS5 overexpression could rescue podosome formation in the absence of TKS4. However, it could not restore ECM degradation. Thus, the two TKS proteins seem to have overlapping functions in filamentous actin formation, and the upregulation of TKS5 expression can substitute for TKS4 in this process [2]. TKS5 has also been implicated in regulating proper MT1-MMP cell surface expression by controlling its exocytosis [85]. At the end of the podosome formation process (and depending on the cell type), single, clustered, ring-like, belt-like, or rosette-like podosomes form with localized MMPs, imparting the cells with motility and ECM-remodeling activities [99].

Invadopodium assembly is very similar to podosome assembly [80]. However, there are a few differences. While podosomes usually contain an actin-rich core surrounded by a vinculin/paxilin adhesion ring, this organized ring structure is absent in invadopodia [100]. NCK1 and GRB2, two TKS-interacting partners (Table 1), have been shown to be present in podosomes and invadopodia. However, their localization patterns are different in the two structures [101]. According to Oser et al., the adaptor protein NCK1 is specifically restricted to invadopodia while GRB2 functions mainly in podosome-like structures and not in the invadopodia of metastatic cells [101]. It is likely that the two degradative cell compartments use distinct mediators for N-WASP recruitment to the site of action [93,96]. For example, in invadopodia, TKS5 might recruit N-WASP via NCK1, while TKS5 interacts with GRB2 during podosome assembly [29,52]. This feature might explain how the different invasive structures can both assemble using the same scaffold molecule.

Besides endowing the cell with an invading phenotype and coordinating cell motility, podosomes might also play a role in cell-cell communication. It was demonstrated that the fifth SH3 domain of TKS5 associates with the intracellular tail of certain ADAM (a disintegrin and metalloprotease) protein family members (Table 1b). TKS5 and ADAM 12, 15, and 19 co-immuno-precipitate and co-localize at podosome sites in SRC-transformed fibroblasts [6]. Although only in a GST pull-down experiment, TKS4 was also reported to bind ADAM15 with its fourth SH3 domain (Table 1a) [34]. An interesting feature of these membrane-localized proteases is that they can act as sheddases [102]. In fact, ADAM proteins are involved in the growth factor or ligand activation by cleaving the inactive, membrane-anchored forms of these molecules to release the active forms, which has been demonstrated in the case of an insulin-like growth factor-binding protein (IGF-BP) [103], Delta-like ligand 1 (DLL1) [104], E-cadherin [105], amphiregulin [106], heparin-binding EGF-like growth factor (HB-EGF) [107], transforming growth factor alpha (TGFα), EGF [108], or tumor necrosis factor alpha (TNFα) [109]. After cleavage by ADAMs, the released cytokines can act on the same, adjacent, or distant cells to allow the “signal sender” cell to communicate with “receiver” cells.

Based on these observations, we propose that TKS proteins might be involved in diverse cell fate-determining mechanisms via podosome organization.

### 3.3. Significance of TKS5 in Invasiveness

TKS5 has been described in several studies as an invadopodium (and podosome) marker [110,111,112,113,114,115,116,117,118], as it is not found in other types of protrusions and adhesive motility structures [80]. Elevated expression levels of the protein have been reported in a number of cancer types [119,120]. As already discussed, TKS5 is involved in the regulation of invadopodium formation and is also known as a key player in metastasis-related processes [85,121]. So far, elevated TKS5 expression has been demonstrated in lung adenocarcinoma [122], glioblastoma cells [119], breast cancer, melanoma cells [120], and keratocystic odontogenic tumor samples [123], where it is primarily correlated with the invasive phenotypes. In addition, upregulated TKS5 expression has been linked to a tissue-invasive, hypermobile pro-inflammatory T cell phenotype in a rheumatoid arthritis model [124]. Analogous to metastasis formation in vivo, it has been shown that altered TKS5 expression can influence the invasive properties of tumor cell lines in vitro [86]. In lung adenocarcinoma, it was also demonstrated that the tumor cell invadopodium activity and metastatic behavior depended on the TKS5 isoform type present [122]. The TKS5 protein has three isoforms (molecular weights of ~150 kDa, ~140 kDa, ~130 kDa) generated via alternative promoter usage as a result of intron 5 retention [125]. Li et al. found that the expression level of the long TKS5 isoform was elevated in metastasis-derived cells when compared with its level in non-metastatic tumor cells [122]. Moreover, a higher TKS5 long/TKS5 short isoform ratio induces invadopodium formation and mediates the development of an invasive phenotype. The functional differences between the isoforms depend on the fact that both transcripts encode five SH3 domains, while the PX domain is missing from the short TKS5 isoform. This short TKS5 cannot organize invadopodium assembly as effectively as the long isoform due to the lack of proper PX domain-dependent membrane localization [122]. Although TKS4 also has several isoforms (~75 kDa, ~90 kDa, ~120 kDa, and ~160 kDa) [46], such a biased isotype preference in invadopodia formation has not been reported.

Metastatic cancer cells cross the basement membrane using MMPs enriched in the invadopodium machinery [126]. In addition to degrading the ECM, the proteolytic activity of ADAM proteases might also facilitate the release of ECM-bound tumor-supporting factors (e.g., EGF and TNFα) and maintain the invasive ability of cancer cells [127,128]. In the context of this special type of tumor cell-extracellular environment communication, invadopodium development and exosome formation were described as connected processes [129]. Exosomes are secreted membrane vesicles that contain a cargo of proteins, mRNAs, and miRNAs highly specific to the “exosome-sender” cell [130]. In terms of cancer, exosomes secreted by tumor cells help establish a tumor-promoting niche via the release of angiogenic and survival factors. It has been demonstrated that TKS5 inhibition in the context of invadopodium formation also greatly decreases exosome formation in a carcinoma cell line [129]. Consistent with this observation, the existence of invadopodia might be an important determining step in exosome formation, representing a newly described cell communication method involved in cancer progression.

### 3.4. The Possible Role of TKS4 and TKS5 in the Compartmentalization of Oxidative Processes

**Figure 3:**
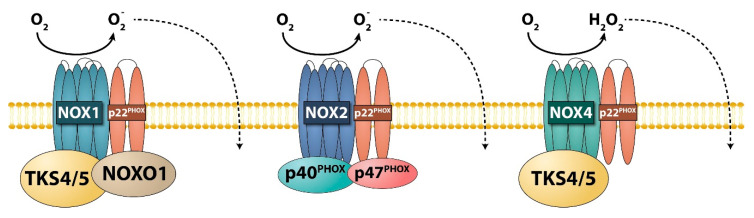
Assembly of NADPH oxidase multi-protein enzyme complexes with the P47 organizer family members. The core of the intracellular enzyme is formed via an interaction between a specific NOX oxidase and p22^phox^ accompanied by activity-modulating organizers such as PX-SH3-structured proteins (NOXO1, TKS4/5, p47^phox^, p40^phox^). The extra-membrane-produced O_2_ is rapidly transformed into H_2_O_2_ and can passively diffuse through the cell-membrane. (Reviewed in Reference [144]).

In general, reactive oxygen species (ROS), including hydrogen peroxide (H_2_O_2_), superoxide anion (O^2-^), and hydroxyl radical (˙OH), are generated as by-products of normal metabolic processes [131]. These molecules act as secondary messengers in normal and pathological cells in which they orchestrate various biological processes [131,132]. However, at high concentrations, they can potentially damage vital signaling molecules and the genome [133]. At specific ROS concentrations and under biological control [134], several ROS-dependent processes and signaling pathways exist, including angiogenesis [135], Notch and Wnt stem cell fate determining signaling [136], the anti-microbial function of phagocytes [137], and pain processing within the nociceptive system [138]. But, how can molecules as simple as ROS modulate such diverse pathways? The best way to answer this question is to briefly summarize the regulation of NADPH oxidase (NOX) transmembrane enzyme complexes (i.e., NOX1, NOX2, NOX3, NOX4, NOX5, DUOX1, and DUOX2) as natural ROS sources [139]. At the molecular level, ROS-generating enzyme-complexes comprise a catalytic subunit (one of the NOX family oxidases), an activity providing subunit (p22^phox^), and several regulatory cytosolic proteins (e.g., members of the p47^phox^ protein family) (Figure 3) [140,141]. Each mammalian NADPH oxidase has a distinct tissue-specific expression pattern [142]. To precisely channel ROS production to the intended targets and to achieve ROS-pathway specificity, NADPH oxidase activity must be compartmentalized within the cells and restricted to spatial cellular microdomains [138,143].

Three members of the p47 organizer protein family (i.e., p47^phox^, p40^phox^, and NOXO1) have well-known functions in the regulation of oxidative processes [145]. It was suspected that TKS proteins, which are members of the same family, can also play regulatory roles in channeling and localizing the NOX enzymes to the podosome/invadopodium membrane to locally increase the ROS concentration [65,146]. TKS5 has been shown to colocalize with ROS [147]. Furthermore, Diaz et al. showed that TKS5 associates with p22^phox^ and the TKS proteins can be involved in processes involving both the NOX1-based and NOX4-based enzyme complexes [65]. In an accompanying paper, Gianni et al. demonstrated that, in a colon cancer cell line, TKS4 recruits the NOX1 NADPH oxidase to the sites of invadopodia and allows ECM degradation [42]. These results raised the possibility that the modulation of ROS levels plays a regulatory role in a subcellular compartment of invadopodia [4,43,146].

It has already been reported that, in the presence of ROS, redox-sensitive cysteine residues in several proteins become oxidized, demonstrating that the conformation of certain enzymes can be changed in an ROS-dependent manner [148]. This remodelling can lead to altered three-dimensional structures in the target proteins that might also alter their catalytic activity [149]. The most highly studied ROS-targeted enzymes are phosphatases (PTPases). When PTPases are inhibited by ROS-dependent cysteine modifications at a site in the catalytic domain, they cannot dephosphorylate their substrate proteins. For example, SRC kinase dephosphorylation in invadopodia is known to be regulated by this mechanism [150]. In an interesting model of invadopodium turnover, the NADPH oxidase regulated by the TKS scaffold proteins produces ROS near the cell-membrane, leading to PTPase inactivation via cysteine modification. The inactivated PTPase then primes the sustained activation of phosphorylated SRC kinase [65]. This process might lead to extended activation of all SRC substrates, including the TKS proteins. At this point in invadopodium development, the TKS scaffold proteins can recruit actin-organizing complexes to the sites of membrane protrusion to stabilize the invadopodium. It is tempting to speculate that the concerted action of the TKS proteins is central in controlling invadopodium turnover via ROS-dependent phosphatase inactivation and coordination of distinct intracellular signaling.

To avoid the harmful side-effects of free radicals, cells control ROS levels by maintaining a balance between ROS production and elimination [151,152]. TKS proteins, as regulators of NOX localization, participate in this process by regulating ROS compartmentalization. Moreover, via their SH3 domains, they facilitate the recruitment of actin cytoskeleton modifiers and ECM degrading machinery in invadopodia [133]. Future studies are needed to determine whether cooperation between the TKS molecules and the NOX complex is also involved in podosome and invadopodium formation in vivo.

### 3.5. Absence of TKS4 Induces Epithelial-Mesenchymal Transition (EMT) and Promotes Invasive Behavior

A novel function of TKS4 in EMT-like processes has been recently discovered by Szeder and colleagues [153]. During EMT, epithelial cells lose epithelial features and functions. The expression levels of E-cadherin, claudins, occludins, and α6β4 integrins are reduced. Thus, cells lose apicobasal polarity and cell-cell attachment ability. This loss of epithelial characteristics is concomitant with the gain of mesenchymal-like features, including N-cadherin, vimentin, fibronectin, β1 and β3 integrins, and MMP expression, as well as the acquisition of motility and invasive properties. These changes are governed by the EMT-inducing transcription factors zinc finger E-box-binding homeobox (ZEB), Snail, and Twist, which inhibit expression of genes responsible for epithelial characteristics and activate expression of mesenchymal-associated genes [154,155]. EMT occurs at certain stages of developmental processes and wound-healing and is an important mechanism in cancer progression from tumor initiation to metastasis and colonization [154,156]. According to Szeder et al., TKS4 knockout (KO) HCT116 colon cancer cells showed a mesenchymal morphology with increased motility and decreased cell-cell adhesion. Loss of E-cadherin and apicobasal polarity was observed together with increased fibronectin and Snail2 transcription factor expression, indicating a shift from an epithelial to mesenchymal-like phenotype. Furthermore, decreased spheroid forming capacity and increased invasiveness in collagen matrix were also observed [153]. The exact mechanism for how the absence of TKS4 may induce EMT in these cells remains unknown. However, two cellular processes known to be influenced by TKS4, i.e., EGFR signaling [157,158] and ROS balance [159,160], have been implicated in affecting EMT in various cancer model systems (see above). EGFR signaling is a known inducer of EMT, causing increased Snail2/Slug and ZEB1 levels and decreased E-cadherin levels [157,158]. Thus, changes in EGFR or ROS signaling in the absence of TKS4 might cause these cancer cells to shift into EMT. Szeder et al. hypothesized that a temporary loss of TKS4 could negatively affect podosome-related cell migration (as described earlier), while the prolonged effect of the absence of the molecule could be increased invasiveness by activating an EMT-like program. They also concluded that the molecule might act differently in cells of epithelial origin (like HCT116) than in those of mesenchymal origin [153]. Despite these interesting findings on the role of TKS4 in EMT processes, more studies are needed to confirm the results of Szeder et al. and to elucidate the exact mechanisms behind this phenomenon.

EMT, along with the reverse process known as mesenchymal-epithelial transition (MET), are crucial processes involved in embryonic development during which they facilitate body formation and tissue and organ differentiation. EMT is involved in such central developmental processes as gastrulation and neural crest cell formation, somitogenesis, cardiac morphogenesis, and trophoblast invasion, which affects placental development, among others [161,162]. A disturbance in EMT (or in MET) can, therefore, have diverse developmental consequences that could also explain the phenotype observed in patients [163,164,165] and mouse models [34,164,166] that lack a functional TKS4 protein.

### 3.6. Cell Differentiation Modulated by TKS Molecules

Cellular differentiation is a fundamental process throughout the lifetime of an organism. During embryogenesis, the spectrum of cells comprising the tissues and organs, which perform a vast number of functions, are derived from a single zygote via differentiation and proper localization in the developing organism via migration [167,168]. During the lifetime of tissues and organs, cells lost through injury or normal cell turnover must be continuously replaced via differentiation from tissue-resident stem and progenitor cells [169,170]. The role of TKS proteins in such contexts will be discussed in this section.

The first observation regarding the role of the TKS proteins in cell fate determination came from Hishida et al. They reported that TKS4 expression is necessary in the early phase of adipogenesis for the expansion and commitment toward adipocytes [12]. By using the 3T3-L1 mouse cell line as an in vitro adipocyte differentiation model in conjunction with a TKS4-silenced derivative, they found that TKS4 down-regulation impaired adipocyte differentiation. (This study was performed before the detailed description of TKS4. Therefore, the original name of the protein, Fad49, was based on its newly identified function, i.e., factor for adipocyte differentiation 49.)

A recent analysis of bone marrow-derived mesenchymal stem/stromal cells (MSCs) revealed a central role of TKS4 in the adipogenesis and osteogenesis of MSCs [166]. These cells serve as common precursors of adipocytes and bone-forming osteoblasts (among others) [171]. During adipogenic or osteogenic induction of mouse MSC cultures, differentiating TKS4 KO cells failed to accumulate lipid droplets or deposit calcium-containing minerals, respectively. Analysis of the expression levels of lipid-regulated genes during adipogenic induction revealed reduced or delayed levels of adipogenic transcription factors, genes driving lipid droplet formation, and sterol and fatty acid metabolism in TKS4 KO cultures [166]. Furthermore, PPARγ2, which is a key transcription factor and regulator of adipose tissue expansion [172], showed no detectable expression at the protein level in TKS4 KO MSC cultures [166]. Upon osteogenic induction, the key osteogenesis-driving transcription factors RUNX2 and osterix showed highly reduced expression levels in TKS4 KO cultures accompanied by reduced bone-forming capacity when compared to wild-type MSCs [166]. Related to this topic, Vas et al. published interesting findings by studying the adipogenic potential of adipose tissue-derived stromal vascular fraction cells. These cell populations also contain MSCs. However, no difference was found between the adipogenic and osteogenic differentiation potential of cells isolated from TKS4 KO and wild-type mice [173]. Perhaps the complexity of the adipose tissue microenvironment (MSCs, preadipocytes, vascular endothelial cells, pericytes) together with the ECM can rescue the differentiation defects of TKS4 KO MSCs or preadipocytes (e.g., 3T3-L1 cells), which fail to properly differentiate in pure in vitro cultures.

It was hypothesized by Oikawa et al. that TKS5 might have an effect on cell-cell fusion. In two studies, TKS5 expression was found to be induced during the course of osteoclast development [19,60]. Osteoclast precursor cells developed podosome-like structures characterized by TKS5 enrichment during the multinucleation process. RNAi-mediated knockdown of TKS5 markedly reduced podosome formation in maturing monocytes (the precursors of osteoclasts) and abolished cell-fusion. The authors also suggested that TKS5, as a master regulator of invadopodium formation, might mediate the fusion-competent protrusion generation necessary for bone metastasis.

TKS scaffold proteins have an instructive effect not only on cell specialization in adult organisms but also during embryonic development. The morphogenic effects of TKS5 were studied by Murphy et al. and Cejudo-Martin et al. in zebrafish [174] and mouse [125] embryos, respectively. TKS5 was found to be necessary in neural crest patterning in zebrafish. TKS5-morpholino zebrafish have cardiac failure, abnormal craniofacial structures, and melanophores with decreased pigmentation. These morphological defects might be explained by reduced neural cell migration during embryonic development due to abnormal podosome-like structure formation in neural stem cells [174]. TKS5 gene-trapped mice are born with a complete cleft of the secondary palate and die shortly after birth [125]. Since trophoblast podosome formation is important in trophoblast function and implantation, and because TKS4 and TKS5 have been implicated in influencing podosome formation, the question arose whether the absence of TKS proteins causes lethality before or after implantation. By genotyping E3.5 pre-implantation blastocysts from TKS4-TKS5 double heterozygous intercross matings, Cejudo-Martin et al. found adequate amounts of double-null blastocysts, suggesting a post-implantation role for TKS5 in mammalian development [125]. These results reveal that the influence of TKS proteins seems to extend to several steps and processes involved in differentiation and embryonic development.

### 3.7. Role of TKS4 in Tissue Homeostasis

As described in the previous section, TKS proteins seem to have determining roles in the cell specialization processes of tissues. A role of TKS4 has also been implicated in the homeostasis of mature adipose and bone tissue. A genome-wide association scan on obesity in a large US Caucasian population found a strong link between body mass index and the chromosomal region of 5q35 with the *Sh3pxd2b* gene [175], supporting the idea that TKS4 has a role in adipose tissue development and/or regulation. No similar association was found by Vogel et al. based on a dataset from children and adolescents [176]. These contradictory results most likely reflect the multifactorial nature of obesity. The development of obesity is dependent on alterations in the composition of the adipose depots [177].

A recent study revealed that TKS4 KO mice had a disturbed adipose depot phenotype involving the beige-ing/browning of white adipose tissue (WAT) depots and a concomitant “whitening” of brown adipose tissue (BAT) [173]. WAT was found to be enriched with smaller and more multi-locular adipocytes and showed higher expression of uncoupling protein 1 (UCP1) at both the RNA and protein levels in tissue samples of TKS4 KO mice compared with the same features in wild-type samples [173]. UCP1 is a marker of brown and beige adipocytes, which are responsible for uncoupling the mitochondrial respiratory chain to stimulate heat production instead of ATP generation [178]. UCP1 showed increased expression in TKS4 KO WAT, indicating white adipocyte beige-ing/browning [173]. The reverse trend was true for the BAT of TKS4 KO mice, which showed an increased adipocyte size with fewer multilocular cells and reduced UCP1 expression, indicating BAT “whitening” or impaired BAT function. A more detailed analysis of one WAT depot showed a shift in the expression patterns of PPARγ-regulated adipogenesis-related genes (e.g., downregulation of the PPARγ target genes *Cebpd*, *Lpl*, *Lipe*, and *Adipoq* and upregulation of the beige transcription factors *Prdm16* and *Ppargc1a*), favoring beige-ing and highlighting PPARγ as a central regulator through which TKS4 can exert its effect on adipocyte homeostasis [173]. Based on these results, TKS4 emerged as an organizer molecule of adipocyte homeostasis-regulating signaling networks.

Studies of the bone structure of a patient with a defective TKS4 gene and TKS4 KO mice revealed altered trabecular systems with increased trabecular separation and porosity resembling an osteoporotic phenotype [179]. Osteoporosis arises from dysregulated bone tissue remodeling when the fine-tuned balance between bone formation and bone resorption is disturbed [180], even though the exact mechanisms of osteoporotic processes are still under investigation. Vas et al. demonstrated that the osteoporotic-like phenotype in TKS4 KO mice did not arise due to an increased osteoclast number or activity and that it likely arose instead due to defective osteoblast differentiation and activity [179]. As it has been mentioned above, TKS4 was found to be indispensable in the differentiation of bone marrow MSCs into functioning osteoblasts [166]. The higher TKS4 expression levels in the immature cell type-enriched fraction of the bone marrow and its presence throughout the differentiation process of osteoblasts as they arise from their precursors (bone-marrow MSCs) highlight the importance of the molecule in bone differentiation [179]. TKS4 was also shown to affect the levels of bone formation markers, i.e., decreased RUNX2 expression in KO bone tissue and reduced osteocalcin levels in TKS4 KO bone marrow, suggesting a role of TKS4 in osteoporotic processes and bone homeostasis [179].

## 4. TKS4- and TKS5-Related Pathological Conditions and Mouse Models

### 4.1. Pathological Conditions Related to TKS Protein Dysfunction

**Figure 4:**
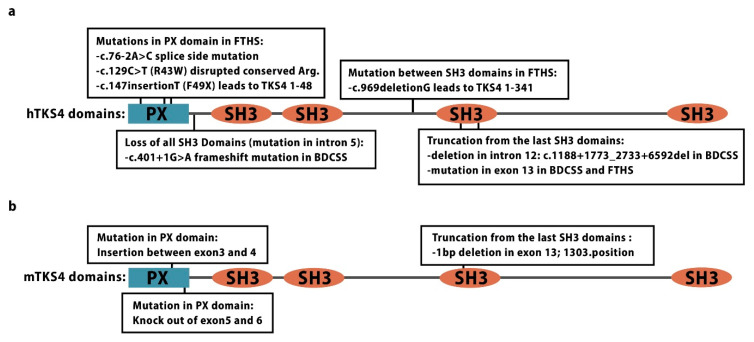
Summary of the location of already documented mutations in human TKS4 gene of patients and mouse TKS4 gene of TKS4 KO strains. (**a**) Mutations along the hTKS4 protein in Frank-ter Haar syndrome (FTHS) and in FTHS-like Borrone dermato-cardio-skeletal syndrome (BDCSS) individuals. The exact sequence location of the mutations in hTKS4 is numbered and the nucleotide deletions, substitutions, or insertions are depicted in black boxes. Iqbal et al. and Bendon et al. published the five mutations connected to FTHS [164,165]. The two BDCSS-associated mutations are described by Wilson et al. [186]. (**b**) Mutations along the mTKS4 protein in the three existing TKS4 KO mouse strains are depicted in black boxes [34,164,166]. PX–Phox homology domain, SH3–SRC homology 3 domain.

Frank-ter Haar syndrome (FTHS, OMIM:249420) is a rare autosomal recessive disease described and named by two groups in the 1970s [181,182]. Most families affected by FTHS have documented consanguinity, and most affected individuals carry a homozygous mutation in the TKS4 gene (*Sh3pxd2b*) on chromosome 5q35.1 [164,165]. FTHS is characterized by craniofacial abnormalities, including a wide anterior fontanel, prominent eyes, and dental anomalies. Other skeletal malformations, including bowing and shortened long bones and kyphosis, are often associated with FTHS, and the most fatal consequences of the disease are cardiac anomalies caused by valve or septal defects. Genome analysis of FTHS patient samples uncovered several major mutations in the TKS4 region, including mutations in the PX domain and between the second and third SH3 domains as well as an extensive deletion from exon 13 that leads to a truncated TKS4 protein/gene product with only two SH3 domains (Figure 4a) [164,165,183,184]. Early stop codon-introducing homozygous mutations (c.147insT or F49X) or a deletion (c.969delG), which lead to the expression of truncated TKS4^1-48^ and TKS4^1-341^ mutant proteins, respectively, were detected in some FTHS-affected families (Figure 4a) [164,185]. In transfected cells, the truncated mutant TKS4^1-48^ protein showed no expression, while TKS4^1-341^ abnormally accumulated in the nucleus, suggesting that these mutations result in dysfunctional TKS4 proteins that could lead to FTHS [5]. Recently, another two mutations in the TKS4-encoding gene (in intron 5 and exon 13) have been linked to an FTHS-related phenotype (Figure 4a) called Borrone dermato-cardio-skeletal syndrome (BDCSS), which causes symptoms such as a coarse face, broad forehead, broad nasal bridge, hypertelorism, megalo-cornea, glaucoma, osteopenia, kyphoscoliosis, and mitral valve prolapse [186]. Despite having an intact TKS4 gene, individuals presenting typical FTHS clinical symptoms are thought to have mis-regulated TKS4 expression at the protein level [164]. The exact mechanism by which mutant TKS4 proteins cause the FTHS symptoms is not known. One suspected mechanism for FTHS development is based on observations of Bögel et al. [11]. In their study, an R43W substitution (c.129C>T) was introduced into the wild type TKS4 protein. This mutation is present in one of the affected FTHS families, and it is located in the conserved region within the PX domain (which is involved in lipid-binding) of the p47 organizer family members [6,187]. The R43W-mutant TKS4 failed to localize to the plasma membrane and was presumably misfolded [11]. Ádám et al. demonstrated that the accumulation of the R43W-mutant TKS4 in aggresomes (at the juxtanuclear region of cells via the microtubule network) is associated with loss of function [5]. These results suggest that the R43W-mutant TKS4 protein might also show similar functional defects in FTHS patients.

Another pathological condition that might be related to a TKS4-dependent process is glaucoma [188]. Although glaucoma is not considered a major diagnostic criteria for FTHS, several FTHS patients suffer from it [163]. This observation led to the hypothesis that TKS4 also has a role in eye development. Analysis of 178 patients with three different forms of glaucoma revealed that the TKS4 gene might harbor rare variants that could affect the pathophysiology of glaucoma. Moreover, TKS4 was present in several ocular cell types important in disease development, reinforcing the possible pathogenic role of the TKS4 variants [189].

In addition to the well-defined function of TKS5 in tumor progression, TKS5 has also been implicated in Alzheimer’s disease-related amyloid-β (Aβ) peptide-mediated neurotoxicity [190]. Malinin et al. demonstrated that ADAM12, a TKS5 binding partner, shows reduced expression in diseased brain samples. Furthermore, they provided evidence that TKS5 is phosphorylated in cultured human neuronal cells exposed to the toxic Aβ protein, resulting in a TKS5-ADAM12 interaction and, ultimately, ADAM12 self-cleavage [191]. Another recent study described a potential role for TKS5 in another pathological condition known as pre-eclampsia [192]. Analysis of affected and healthy placentas led to the identification of two upregulated factors, i.e., leptin and TKS5. Although the methylation pattern of the TKS5 promoter region was not significantly altered, one CpG island in the gene body showed higher methylation. The authors propose that TKS5, which is a major organizer of podosome formation, might be involved in trophoblast cell migration in the placenta. As a consequence, impaired TKS5-dependent pathways in trophoblast cells might lead to pre-eclampsia [193].

### 4.2. Knockout Mouse Models

The first TKS4 KO mouse line arose spontaneously in the 51st generation of a B10.A-H2h4/(4R)SgDvEg mouse strain [34]. The mutant mice were called “nee mice” based on a distinct phenotype, i.e., “nose-ear-eye” deformities. A genome analysis showed that the nee mice carry a 1-bp deletion (which introduces a frameshift mutation) in exon 13 of the *Sh3pxd2b* gene (Figure 4b) [34]. Iqbal et al. has generated another mouse line with a mixed genetic background carrying an insertion between exons 3 and 4 of the *Sh3pxd2b* gene on chromosome 11 [164] (Figure 4b). Recently, an FTHS mouse model has been reported in a C57Bl/6 background by Dülk et al. In these TKS4^-/-^ mice, TKS4 was knocked out by introducing an insertion between exons 5 and 6 of the *Sh3pxd2b* gene (Figure 4b) [166]. All of these TKS4-mutant strains show very similar phenotypes that are reminiscent of the clinical symptoms of FTHS. These phenotypes include a shorter nasal bone, an overall decreased size, and a tendency to develop early onset glaucoma in their enlarged/prominent eyes [34,164,166,179]. Moreover, cardiac examination of the artificially generated TKS4 KO mice revealed variable deficiencies, including septal and mitral valve defects remarkably similar to those of FTHS patients [164]. Skeletal abnormalities, e.g., kyphosis [34,164,166], and reduced bone mineral density [34,179], which are both reminiscent of FTHS features, have also been described in the TKS4-mutant mice. Lipodystrophy is a general feature characterized by highly reduced visceral WAT mass in the case of TKS4 null mice [34,173], even though a low amount of subcutaneous fat tissue has only been reported in one diseased patient [164].

**Figure 5:**
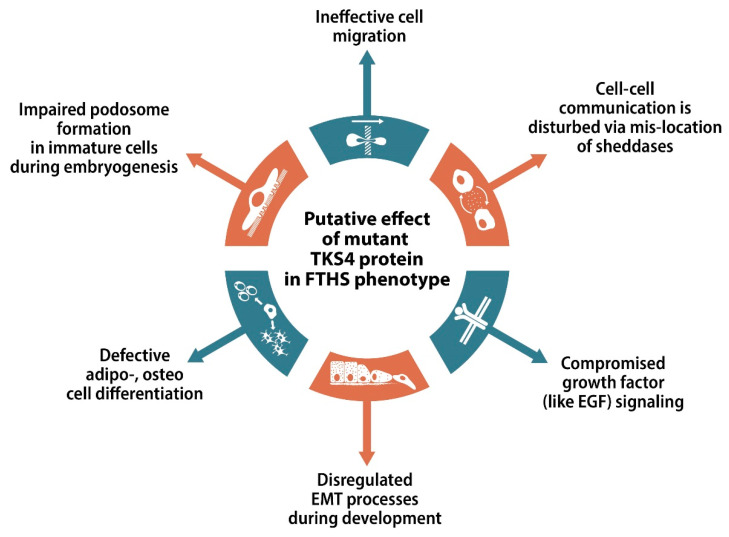
Putative role of mutated TKS4 in Frank-ter Haar syndrome (FTHS). During embryogenesis, differentiating cells must react properly to growth factor signals, migrate via podosome machinery to the determined position in the embryo, degrade the ECM during their migration, send extracellular signaling molecules to other cells, and occasionally undergo EMT. In each step, TKS4 has an experimentally described role. However, there are insufficient evidence at the organismal, tissue, cellular, and even biochemical levels to have a clear understanding of how TKS4 participates in disease manifestation. ECM–extracellular matrix, EMT–epithelial-mesenchymal transition.

Taken together, the abnormalities observed in TKS4 KO mouse lines support the hypothesis that the presence of a mutant TKS4 gene has a role in FTHS. The exact detailed mechanism by which mutations in TKS4 cause such a diverse range of phenotypes in patients is still unknown. A few putative mechanisms are summarized in Figure 5. TKS4 has its highest expression levels in embryonic tissues [2], suggesting that the most notable phenotype-determining effects of the molecule are exerted during embryonic development. Podosome formation and migration of patterning immature cells are tightly linked processes during healthy embryonic development in response to instructive signals [81]. Since TKS4 and TKS5 are key players in functional podosome formation [2,86], mis-regulated podosome assembly early in development in FTHS patients might have a causative role in the manifestation of the related symptoms. Defective endothelial cell motility in complex three-dimensional ECM environments and diminished vessel sprout growth have also been reported in the absence of TKS4, possibly leading to negative effects on tubular heart formation and cardiac development [194]. If the TKS4 scaffold function is absent, EGF-induced cell migration might also be defective [11]. Therefore, TKS4 KO cells might fail to migrate in response to growth factor signal stimulation in general. The involvement of TKS4 in the EMT processes [153] may also affect embryonic development at several developmental stages, including endocardium formation, which is a process involving three consecutive EMT/MET events [161]. Disturbed EMT function could explain why patients and mouse models show defective cardiac development and functionality, expanding the list of processes by which the absence of TKS4 could cause such characteristic gross phenotypes.

Homozygous loss of TKS5 is usually lethal in early neonatal life [125]. Cejudo-Martin et al. created TKS5 KO mice by inserting a trapping vector VICTR 37 between exons 1 and 2 on chromosome 19 in a C57Bl/6Jx129/SvJ mixed background and in a C57Bl/6J pure background. TKS5 gene-trapped (TKS5^trap/trap^) mutant mice of both genetic backgrounds are born at Mendelian ratios, but have a reduced lifespan. In the C57Bl/6Jx129/SvJ background, 50% of the mutant mice have a complete cleft of the secondary palate and die within 24 h after birth, and only 20% reach adulthood with no visible phenotypic defects. Furthermore, 30% of the neonates die between day one after birth and weaning despite having a normal palate. By contrast, in the C57Bl/6J pure background, neonatal mortality and cleft palate incidence rises to 90%. The remaining 10% of animals show no cleft palate but die shortly after birth of unknown causes. The authors concluded that strain purity is a determining factor in the phenotypic manifestation of TKS5 loss. No TKS4^-/-^ and TKS5^trap/trap^ double null mice are born from crosses of heterozygous TKS4 and TKS5 mutant parents, suggesting that the functions of the two molecules can overlap or complement each other during development [125]. Neither TKS4 nor TKS5 mutant animals show gross phenotypic alterations when they are heterozygous for the mutation [34,125,164].

## 5. Conclusions and Future Perspectives

Besides mediating the assembly of signaling components, the scaffold proteins TKS4 and TKS5 have an emerging role as regulated, active facilitators of the crosstalk between multiple signal transduction pathways modulating diverse and complex molecular networks. Future research focusing on the discovery of novel binding partners is expected to reveal not only the detailed molecular level mechanisms resulting in TKS-related phenotypes in FTHS patients and TKS KO animal models, but also, to shed light on yet unknown functions of these proteins. For example, while both TKS4 and 5 were found to be potential binding partners of the Fas ligand (FasL, CD178) (Table 1), which is a known cell death inducer [39,40], possible functions for TKS4/5 in the regulation of cell death have not yet been investigated. A detailed understanding of the TKS4/5 interactome, bolstered by an in-depth description of the molecular modifications of the TKS4/5 proteins during signaling may facilitate the development of new therapies to correct defects in signaling pathways underlying pathological conditions.

## Figures and Tables

**Table 1:** Known protein binding partners of TKS4 and TKS5. The known binding partners of (**a**) TKS4 and (**b**) TKS5 are shown with the methods of detection and the binding sites within the TKS molecules. Some of the well-described functions of the binding partners are also listed. ECM–extracellular matrix, EMT–epithelial-mesenchymal transition, ITC–isothermal titration calorimetry, NOX1–NADPH oxidase 1, PRR–proline-rich region, ROS–reactive oxygen species, RTK–receptor tyrosine kinase. * The first and second SH3 domains cooperate to form a common “super SH3 platform” and allow the binding of the proline-rich region of the partner protein [33].

(**a**)			
**TKS4**			
**Partner**	**Method**	**TKS4-Interacting Site**	**Function**
ADAM15 [34]	GST pull-down assay	4th SH3 domain	Ectodomain shedding, cell adhesion, and signaling [35]
Cortactin [9]	Co-immunoprecipitation, GST pull-down assay, immunofluorescence co-localization	Unknown	Regulation of actin cytoskeleton [36]
CR16 [37]	GST pull-down assay	Weak interaction with the 2nd, 3rd, and 4th SH3 domains	Reorganization of actin cytoskeleton [38]
DNM2 [37]	GST pull-down assay	3rd SH3 domain	Endo-/exocytosis [37]
FasL (CD178) [39]	Phage display screening	3rd and 4th SH3 domains	Apoptosis induction [40]
GRB2 [28]	Affinity purification–selected reaction monitoring mass spectrometry	Unknown	Adaptor protein involved in the regulation of RTK signaling, cycle progression, actin-based cell motility, podosome formation [41]
NOXA1 [42,43]	Co-immunoprecipitation, GST pull-down assay	Unknown	ROS generation through NOX1 activation [44]
N-WASP [37]	GST pull-down assay	2nd SH3 domain	A scaffold protein regulating actin cytoskeleton reorganization, and actin polymerization during cell motility and invasion [45]
OPHN1 [37]	GST pull-down assay	3rd SH3 domain	Endo-/exocytosis [37]
RUK/CIN85 [46]	GST pull-down assay	Unknown	Adaptor protein that recruits endocytotic regulatory proteins, and regulates RTK internalization, trafficking, and degradation [47]
SRC [9,32]	Co-immunoprecipitation; GST pull-down and fluorescence-polaziation assays, Duolink proximity ligation assay	PRR (aa: 466–474); P-Tyr motif (aa: 508–511)	Regulation of cell growth, differentiation, proliferation, survival, adhesion, migration, and motility [9,32]
SYNJ1 [37]	GST pull-down assay	3rd SH3 domain and weak interaction with the 4th SH3 domain	Endo-/exocytosis [37]
(**b**)			
**TKS5**			
**Partner**	**Method**	**TKS5-Interacting Site**	**Function**
ADAM12 [6]	Co-immunoprecipitation, immunofluorescence co-localization	5th SH3 domain	Cell adhesion and fusion, extracellular matrix restructuring, reorganization of actin cytoskeleton, regulation of ectodomain shedding [48]
ADAM15 [6]	Co-immunoprecipitation	5th SH3 domain	Cell adhesion, degradation of ECM components, ectodomain shedding of membrane-bound growth factors [35]
ADAM19 [6]	Phage display screen, co-immunoprecipitation	5th SH3 domain	Extracellular matrix breakdown and reconstruction, ectodomain shedding, role in embryogenesis, cardiovascular system development, obesity, and insulin resistance [49]
β-dystroglycan [50]	Phage display screen, GST pull-down assay, co-immunoprecipitation, immunofluorescence co-localization	3rd SH3 domain	Links the extracellular matrix to the intracellular actin cytoskeleton [50]
CircSKA3 [51]	Co-immunoprecipitation, pull-down assay	Not specified	Circular RNA, an inducer of invadopodium formation [51]
Drebrin [52]	Co-immunoprecipitation	Unknown	An actin-binding protein involved in the regulation of actin filament organization, role in cell migration, cell process formation, intercellular communication, metastasis, and brain development [53]
Dynamin [29,33]	Peptide spot membrane assay, GST pull-down assay, ITC, immunofluorescence co-localization, GST pull-down assay, mass spectrometry/Western blotting	1st and 2nd SH3 domains; 1st and 5th SH3 domains	Regulation of actin cytoskeleton, podosome/invadopodium formation, role in endocytosis [54]
F-actin [29]	GST pull-down assay, and mass spectrometry	5th SH3 domain	Component of cytoskeleton [55]
FasL (CD178) [39]	Phage display screening	5th SH3 domain	Apoptosis induction [40]
FGD1 [56]	Co-immunoprecipitation and mass spectrometry, GST pull-down assay, immunofluorescence co-localization	4th and 5th SH3 domains	A guanine nucleotide exchange factor for the Rho-GTPase CDC42, assembly of podosomes and invadopodia, control of secretory membrane-trafficking, and cell cycle [56,57]
Girdin [58]	Co-immunoprecipitation, immunofluorescence co-localization	Unknown	actin-binding protein regulating actin remodeling and cell polarity, collective migration of neuroblasts, epithelial and cancer cells [59]
GRB2 [28,29]	Co-immunoprecipitation	Polyproline sequences	An adaptor protein involved in cell cycle progression and actin-based cell motility, podosome formation [41]
IRTKS [60]	GST pull-down assay	First binding site located in the segment comprising the 1st and 2nd SH3 domains, second binding site located in the segment comprising the 3rd and 4th SH3 domains	Regulation of plasma membrane dynamics, actin cytoskeleton remodeling, cell migration and polarization, insulin signaling [61]
MT4-MMP [62]	Co-immunoprecipitation	Unknown	Induction of invadopodia and amoeboid movement, degradation of ECM components, role in hypoxia-mediated metastasis [62]
NCK [52]	Co-immunoprecipitation, fluorescence co-localization	Linker region between the 3rd and 4th SH3 domains containing pY557	Adaptor protein involved in cytoskeletal remodeling, invadopodium formation, cell proliferation [63]
Nogo-B [29]	GST pull-down assay and mass spectrometry	5th SH3 domain	Roles in vascular remodeling, cell migration and proliferation, and EMT [64]
NOXA1 [42,43]	Co-immunoprecipitation, GST pull-down assay	One or more of the five SH3 domains	ROS generation through NOX1 activation [44]
N-WASP [29]	GST pull-down assay and mass spectrometry/Western blotting, co-immunoprecipitation	All five SH3 domains	A scaffold protein regulating actin cytoskeleton reorganization, and actin polymerization during cell motility and invasion [45]
p22^phox^ [65]	Co-immunoprecipitation	1st and 2nd SH3 domains	Subunit of NADPH oxidases involved in ROS generation through NOX activity [66]
Rab40b [67]	GST pull-down assay, co-immunoprecipitation,	PX-domain: sites 14-KRR-19 and Y24 in 23-YVYI-28	A GTPase required for the sorting and secretion of MMP2 and MMP9, promotion of migration, invasion, and metastasis of cancer cells [67,68]
RET [69]	Co-immunoprecipitation, immunofluorescence co-localization	Unknown	A receptor tyrosine kinase mediating stress fiber formation, cell polarization, directional migration and invasion, enhancement of proteolytic activity [69]
SOS1 [33]	Immunofluorescence co-localization, peptide spot membrane assay, GST pull-down assay, isothermal titration calorimetry	1st and 2nd SH3 domains *	A guanine nucleotide exchange factor promoting Ras and Rac activation downstream of a variety of receptors such as RTKs [70]
Tubulin [29]	GST pull-down assay and mass spectrometry	3rd SH3 domain	Component of microtubules, affects cell division, differentiation, intracellular transport, motility [71]
WIP [29]	GST pull-down assay and mass spectrometry	3rd and 5th SH3 domains	Regulation of actin cytoskeleton assembly and remodeling [72]
XB130 [73]	Yeast two-hybrid screening, co-immunoprecipitation, GST pull-down assay, immunofluorescence co-localization	5th SH3 domain	A scaffold protein influencing cell growth, survival, and migration [73]
Zyxin [29]	GST pull-down assay and mass spectrometry	3rd and 5th SH3 domains	A focal adhesion protein involved in actin cytoskeleton assembly [74]

## Abbreviations

ADAM	a disintegrin and metalloproteinase
BAT	brown adipose tissue
BDCSS	Borrone dermato-cardio-skeletal syndrome
ECM	extracellular matrix
EGF	epidermal growth factor
EMT	epithelial-mesenchymal transition
FTHS	Frank-ter Haar syndrome
GST	glutathione S-transferase
H_2_O_2_	hydrogen peroxide
ITC	isothermal titration calorimetry
KO	knockout
MET	mesenchymal-epithelial transition
miRNA	microRNA
MMP	matrix metalloproteases
mRNA	messenger RNA
MSCs	mesenchymal stem/stromal cells
nee mice	“nose-ear-eye” deformities mice
NOX	NADPH oxidase
O^2-^	superoxide anion
˙OH	hydroxyl radical
PI(4,5)P2	phosphatidylinositol (4,5)-bisphosphate
PI(3,4,5)P3	phosphatidylinositol (3,4,5)-trisphosphate
PPARγ	peroxisome proliferator-activated receptor gamma
PRR	proline-rich region
PTPases	phosphatases
PX domain	Phox homology domain
ROS	reactive oxygen species
RTK	receptor tyrosine kinase
SH3	SRC homology 3 domain
TKS	tyrosine kinase substrate
UCP1	uncoupling protein 1
WAT	white adipose tissue

## References

[1] Buday L., Tompa P. (2010). Functional classification of scaffold proteins and related molecules. FEBS J..

[2] Buschman M.D., Bromann P.A., Cejudo-Martin P., Wen F., Pass I., Courtneidge S.A. (2009). The Novel Adaptor Protein Tks4 (SH3PXD2B) is Required for Functional Podosome Formation. Mol. Biol. Cell.

[3] Lock P., Abram C.L., Gibson T., Courtneidge S.A. (1998). A new method for isolating tyrosine kinase substrates used to identify Fish, an SH3 and PX domain-containing protein, and Src substrate. EMBO J..

[4] Weaver A.M. (2009). Regulation of Cancer Invasion by Reactive Oxygen Species and Tks Family Scaffold Proteins. Sci. Signal..

[5] Ádám C., Fekete A., Bőgel G., Németh Z., Tőkési N., Ovádi J., Liliom K., Pesti S., Geiszt M., Buday L. (2015). Accumulation of the PX domain mutant Frank-ter Haar syndrome protein Tks4 in aggresomes. Cell Commun. Signal..

[6] Abram C.L., Seals D.F., Pass I., Salinsky D., Maurer L., Roth T.M., Courtneidge S.A. (2003). The Adaptor Protein Fish Associates with Members of the ADAMs Family and Localizes to Podosomes of Src-transformed Cells. J. Biol. Chem..

[7] Kurochkina N., Guha U. (2013). SH3 domains: Modules of protein–protein interactions. Biophys. Rev..

[8] Kawahara T., Lambeth J.D. (2007). Molecular evolution of Phox-related regulatory subunits for NADPH oxidase enzymes. BMC Evol. Biol..

[9] Lányi Á., Baráth M., Péterfi Z., Bőgel G., Orient A., Simon T., Petrovszki E., Kis-Tóth K., Sirokmány G., Rajnavölgyi É. (2011). The Homolog of the Five SH3-Domain Protein (HOFI/SH3PXD2B) Regulates Lamellipodia Formation and Cell Spreading. PLoS ONE.

[10] Daly C., Logan B., Breeyear J., Whitaker K., Ahmed M., Seals D.F. (2020). Tks5 SH3 domains exhibit differential effects on invadopodia development. PLoS ONE.

[11] Bögel G., Gujdár A., Geiszt M., Lányi Á., Fekete A., Sipeki S., Downward J., Buday L. (2012). Frank-ter Haar Syndrome Protein Tks4 Regulates Epidermal Growth Factor-dependent Cell Migration. J. Biol. Chem..

[12] Hishida T., Eguchi T., Osada S., Nishizuka M., Imagawa M. (2008). A novel gene, fad49, plays a crucial role in the immediate early stage of adipocyte differentiation via involvement in mitotic clonal expansion. FEBS J..

[13] Teasdale R.D., Collins B.M. (2012). Insights into the PX (phox-homology) domain and SNX (sorting nexin) protein families: Structures, functions and roles in disease. Biochem. J..

[14] Yaffe M.B. (2002). The p47phox PX Domain: Two Heads Are Better Than One!. Structure.

[15] Groemping Y., Lapouge K., Smerdon S.J., Rittinger K. (2003). Molecular Basis of Phosphorylation-Induced Activation of the NADPH Oxidase. Cell.

[16] Belambri S.A., Rolas L., Raad H., Hurtado-Nedelec M., Dang P.M.-C., El-Benna J. (2018). NADPH oxidase activation in neutrophils: Role of the phosphorylation of its subunits. Eur. J. Clin. Investig..

[17] El-Benna J., Dang P.M.-C., Gougerot-Pocidalo M.-A., Marie J.-C., Braut-Boucher F. (2009). p47phox, the phagocyte NADPH oxidase/NOX2 organizer: Structure, phosphorylation and implication in diseases. Exp. Mol. Med..

[18] Meijles D.N., Fan L.M., Howlin B.J., Li J.-M. (2014). Molecular Insights of p47phox Phosphorylation Dynamics in the Regulation of NADPH Oxidase Activation and Superoxide Production. J. Biol. Chem..

[19] Oikawa T., Oyama M., Kozuka-Hata H., Uehara S., Udagawa N., Saya H., Matsuo K. (2012). Tks5-dependent formation of circumferential podosomes/invadopodia mediates cell–cell fusion. J. Cell Biol..

[20] Casaletto J.B., McClatchey A.I. (2012). Spatial regulation of receptor tyrosine kinases in development and cancer. Nat. Rev. Cancer.

[21] Wee P., Wang Z. (2017). Epidermal Growth Factor Receptor Cell Proliferation Signaling Pathways. Cancers.

[22] Belsches A.P., Haskell M.D., Parsons S.J. (1997). Role of c-Src tyrosine kinase in EGF-induced mitogenesis. Front. Biosci..

[23] Alonso G., Koegl M., Mazurenko N., Courtneidge S.A. (1995). Sequence Requirements for Binding of Src Family Tyrosine Kinases to Activated Growth Factor Receptors. J. Biol. Chem..

[24] Bromann P.A., Korkaya H., Courtneidge S.A. (2004). The interplay between Src family kinases and receptor tyrosine kinases. Oncogene.

[25] Sierke S.L., Longo G.M., Koland J.G. (1993). Structural Basis of Interactions Between Epidermal Growth Factor Receptor and SH2 Domain Proteins. Biochem. Biophys. Res. Commun..

[26] Fekete A., Bőgel G., Pesti S., Péterfi Z., Geiszt M., Buday L. (2013). EGF regulates tyrosine phosphorylation and membrane-translocation of the scaffold protein Tks5. J. Mol. Signal..

[27] Gianni D., Taulet N., DerMardirossian C., Bokoch G.M. (2010). c-Src–Mediated Phosphorylation of NoxA1 and Tks4 Induces the Reactive Oxygen Species (ROS)–Dependent Formation of Functional Invadopodia in Human Colon Cancer Cells. Mol. Biol. Cell.

[28] Bisson N., James D.A., Ivosev G., Tate S.A., Bonner R., Taylor L., Pawson T. (2011). Selected reaction monitoring mass spectrometry reveals the dynamics of signaling through the GRB2 adaptor. Nat. Biotechnol..

[29] Oikawa T., Itoh T., Takenawa T. (2008). Sequential signals toward podosome formation in NIH-src cells. J. Cell Biol..

[30] Crimaldi L., Courtneidge S.A., Gimona M. (2009). Tks5 recruits AFAP-110, p190RhoGAP, and cortactin for podosome formation. Exp. Cell Res..

[31] Thapa N., Tan X., Choi S., Lambert P.F., Rapraeger A.C., Anderson R.A. (2016). The Hidden Conundrum of Phosphoinositide Signaling in Cancer. Trends Cancer.

[32] Dülk M., Szeder B., Glatz G., Merő B.L., Koprivanacz K., Kudlik G., Vas V., Sipeki S., Cserkaszky A., Radnai L. (2018). EGF Regulates the Interaction of Tks4 with Src through Its SH2 and SH3 Domains. Biochemistry.

[33] Rufer A.C., Rumpf J., von Holleben M., Beer S., Rittinger K., Groemping Y. (2009). Isoform-Selective Interaction of the Adaptor Protein Tks5/FISH with Sos1 and Dynamins. J. Mol. Biol..

[34] Mao M., Thedens D.R., Chang B., Harris B.S., Zheng Q.Y., Johnson K.R., Donahue L.R., Anderson M.G. (2009). The podosomal-adaptor protein SH3PXD2B is essential for normal postnatal development. Mamm. Genome.

[35] Mattern J., Roghi C.S., Hurtz M., Knäuper V., Edwards D.R., Poghosyan Z. (2019). ADAM15 mediates upregulation of Claudin-1 expression in breast cancer cells. Sci. Rep..

[36] Sharafutdinov I., Backert S., Tegtmeyer N. (2020). Cortactin: A Major Cellular Target of the Gastric Carcinogen Helicobacter pylori. Cancers.

[37] Kropyvko S.V. (2015). New partners of TKS4 scaffold protein. Biopolym. Cell.

[38] Kropyvko S., Gryaznova T., Morderer D., Rynditch A. (2017). Mammalian verprolin CR16 acts as a modulator of ITSN scaffold proteins association with actin. Biochem. Biophys. Res. Commun..

[39] Voss M., Lettau M., Janssen O. (2009). Identification of SH3 domain interaction partners of human FasL (CD178) by phage display screening. BMC Immunol..

[40] Glukhova X.A., Trizna J.A., Proussakova O.V., Gogvadze V., Beletsky I.P. (2018). Impairment of Fas-ligand–caveolin-1 interaction inhibits Fas-ligand translocation to rafts and Fas-ligand-induced cell death. Cell Death Dis..

[41] Giubellino A., Burke J.T.R., Bottaro D.P. (2008). Grb2 signaling in cell motility and cancer. Expert Opin. Ther. Targets.

[42] Gianni D., Diaz B., Taulet N., Fowler B., Courtneidge S.A., Bokoch G.M. (2009). Novel p47phox-Related Organizers Regulate Localized NADPH Oxidase 1 (Nox1) Activity. Sci. Signal..

[43] Gianni D., DerMardirossian C., Bokoch G.M. (2011). Direct interaction between Tks proteins and the N-terminal proline-rich region (PRR) of NoxA1 mediates Nox1-dependent ROS generation. Eur. J. Cell Biol..

[44] Schröder K., Weissmann N., Brandes R.P. (2017). Organizers and activators: Cytosolic Nox proteins impacting on vascular function. Free Radic. Biol. Med..

[45] Hou J., Yang H., Huang X., Leng X., Zhou F., Xie C., Zhou Y., Xu Y. (2017). N-WASP promotes invasion and migration of cervical cancer cells through regulating p38 MAPKs signaling pathway. Am. J. Transl. Res..

[46] Bazalii A.V., Samoylenko A.A., Petukhov D.M., Rynditch A.V., Redowicz M.-J., Drobot L.B. (2014). Interaction between adaptor proteins Ruk/CIN85 and Tks4 in normal and tumor cells of different tissue origins. Biopolym. Cell.

[47] Kong M.S., Hashimoto-Tane A., Kawashima Y., Sakuma M., Yokosuka T., Kometani K., Onishi R., Carpino N., Ohara O., Kurosaki T. (2019). Inhibition of T cell activation and function by the adaptor protein CIN85. Sci. Signal..

[48] Nyren-Erickson E.K., Jones J.M., Srivastava D.K., Mallik S. (2013). A disintegrin and metalloproteinase-12 (ADAM12): Function, roles in disease progression, and clinical implications. Biochim. Biophys. Acta BBA Gen. Subj..

[49] Weerasekera L., Rudnicka C., Sang Q.-X., Curran J.E., Johnson M.P., Moses E.K., Göring H.H.H., Blangero J., Hricova J., Schlaich M. (2017). ADAM19: A Novel Target for Metabolic Syndrome in Humans and Mice. Mediat. Inflamm..

[50] Thompson O., Kleino I., Crimaldi L., Gimona M., Saksela K., Winder S.J. (2008). Dystroglycan, Tks5 and Src Mediated Assembly of Podosomes in Myoblasts. PLoS ONE.

[51] Du W.W., Yang W., Li X., Fang L., Wu N., Li F., Chen Y., He Q., Liu E., Yang Z. (2020). The Circular RNA circSKA3 Binds Integrin β1 to Induce Invadopodium Formation Enhancing Breast Cancer Invasion. Mol. Ther..

[52] Stylli S.S., Stacey T.T.I., Verhagen A.M., Xu S.S., Pass I., Courtneidge S.A., Lock P. (2009). Nck adaptor proteins link Tks5 to invadopodia actin regulation and ECM degradation. J. Cell Sci..

[53] Shirao T., Hanamura K., Koganezawa N., Ishizuka Y., Yamazaki H., Sekino Y. (2017). The role of drebrin in neurons. J. Neurochem..

[54] Zhang R., Lee D.M., Jimah J.R., Gerassimov N., Yang C., Kim S., Luvsanjav D., Winkelman J., Mettlen M., Abrams M.E. (2020). Dynamin regulates the dynamics and mechanical strength of the actin cytoskeleton as a multifilament actin-bundling protein. Nat. Cell Biol..

[55] Grintsevich E.E., Yesilyurt H.G., Rich S.K., Hung R.-J., Terman J.R., Reisler E. (2016). F-actin dismantling through a redox-driven synergy between Mical and cofilin. Nat. Cell Biol..

[56] Zagryazhskaya-Masson A., Monteiro P., Macé A.-S., Castagnino A., Ferrari R., Infante E., Duperray-Susini A., Dingli F., Lanyi A., Loew D. (2020). Intersection of TKS5 and FGD1/CDC42 signaling cascades directs the formation of invadopodia. J. Cell Biol..

[57] Genot E., Daubon T., Sorrentino V., Buccione R. (2012). FGD1 as a central regulator of extracellular matrix remodelling—Lessons from faciogenital dysplasia. J. Cell Sci..

[58] Ke Y., Bao T., Zhou Q., Wang Y., Ge J., Fu B., Wu X., Tang H., Shi Z., Lei X. (2017). Discs large homolog 5 decreases formation and function of invadopodia in human hepatocellular carcinoma via Girdin and Tks5. Int. J. Cancer.

[59] Wang X., Enomoto A., Weng L., Mizutani Y., Abudureyimu S., Esaki N., Tsuyuki Y., Chen C., Mii S., Asai N. (2018). Girdin/GIV regulates collective cancer cell migration by controlling cell adhesion and cytoskeletal organization. Cancer Sci..

[60] Oikawa T., Matsuo K. (2012). Possible role of IRTKS in Tks5-driven osteoclast fusion. Commun. Integr. Biol..

[61] Li L., Liu H., Baxter S.S., Gu N., Ji M., Zhan X. (2016). The SH3 domain distinguishes the role of I-BAR proteins IRTKS and MIM in chemotactic response to serum. Biochem. Biophys. Res. Commun..

[62] Yan X., Cao N., Chen Y., Lan H.-Y., Cha J.-H., Yang W.-H., Yang M.-H. (2020). MT4-MMP promotes invadopodia formation and cell motility in FaDu head and neck cancer cells. Biochem. Biophys. Res. Commun..

[63] Chaki S.P., Barhoumi R., Rivera G.M. (2019). Nck adapter proteins promote podosome biogenesis facilitating extracellular matrix degradation and cancer invasion. Cancer Med..

[64] Zhu B., Chen S., Hu X., Jin X., Le Y., Cao L., Yuan Z., Lin Z., Jiang S., Sun L. (2017). Knockout of the Nogo-B Gene Attenuates Tumor Growth and Metastasis in Hepatocellular Carcinoma. Neoplasia.

[65] Diaz B., Shani G., Pass I., Anderson D., Quintavalle M., Courtneidge S.A. (2009). Tks5-Dependent, Nox-Mediated Generation of Reactive Oxygen Species Is Necessary for Invadopodia Formation. Sci. Signal..

[66] Nagaraj C., Tabeling C., Nagy B.M., Jain P.P., Marsh L.M., Papp R., Pienn M., Witzenrath M., Ghanim B., Klepetko W. (2017). Hypoxic vascular response and ventilation/perfusion matching in end-stage COPD may depend on p22phox. Eur. Respir. J..

[67] Jacob A., Linklater E., Bayless B.A., Lyons T., Prekeris R. (2016). The role and regulation of Rab40b–Tks5 complex during invadopodia formation and cancer cell invasion. J. Cell Sci..

[68] Li Y., Jia Q., Wang Y., Li F., Jia Z., Wan Y. (2015). Rab40b upregulation correlates with the prognosis of gastric cancer by promoting migration, invasion, and metastasis. Med. Oncol..

[69] Lian E.Y., Hyndman B.D., Moodley S., Maritan S.M., Mulligan L.M. (2020). RET isoforms contribute differentially to invasive processes in pancreatic ductal adenocarcinoma. Oncogene.

[70] Gerboth S., Frittoli E., Palamidessi A., Baltanas F.C., Salek M., Rappsilber J., Giuliani C., Troglio F., Rolland Y., Pruneri G. (2018). Phosphorylation of SOS1 on tyrosine 1196 promotes its RAC GEF activity and contributes to BCR-ABL leukemogenesis. Leukemia.

[71] Prassanawar S.S., Panda D. (2019). Tubulin heterogeneity regulates functions and dynamics of microtubules and plays a role in the development of drug resistance in cancer. Biochem. J..

[72] Sokolik C.G., Qassem N., Chill J.H. (2020). The Disordered Cellular Multi-Tasker WIP and Its Protein–Protein Interactions: A Structural View. Biomolecules.

[73] Moodley S., Hui Bai X., Kapus A., Yang B., Liu M. (2015). XB130/Tks5 scaffold protein interaction regulates Src-mediated cell proliferation and survival. Mol. Biol. Cell.

[74] Kotb A., Hyndman M.E., Patel T.R. (2018). The role of zyxin in regulation of malignancies. Heliyon.

[75] Buday L., Downward J. (2007). Roles of cortactin in tumor pathogenesis. Biochim. Biophys. Acta BBA Rev. Cancer.

[76] MacGrath S.M., Koleske A.J. (2012). Cortactin in cell migration and cancer at a glance. J. Cell Sci..

[77] Mader C.C., Oser M., Magalhaes M.A.O., Bravo-Cordero J.J., Condeelis J., Koleske A.J., Gil-Henn H. (2011). An EGFR–Src–Arg–Cortactin Pathway Mediates Functional Maturation of Invadopodia and Breast Cancer Cell Invasion. Cancer Res..

[78] Chen Y.-C., Baik M., Byers J.T., Chen K.T., French S.W., Díaz B. (2019). Experimental supporting data on TKS5 and Cortactin expression and localization in human pancreatic cancer cells and tumors. Data Brief.

[79] Thuault S., Mamelonet C., Salameh J., Ostacolo K., Chanez B., Salaün D., Baudelet E., Audebert S., Camoin L., Badache A. (2020). A proximity-labeling proteomic approach to investigate invadopodia molecular landscape in breast cancer cells. Sci. Rep..

[80] Paterson E.K., Courtneidge S.A. (2018). Invadosomes are coming: New insights into function and disease relevance. FEBS J..

[81] Alonso F., Spuul P., Daubon T., Kramer I., Génot E. (2019). Variations on the theme of podosomes: A matter of context. Biochim. Biophys. Acta BBA Mol. Cell Res..

[82] Linder S., Wiesner C. (2015). Tools of the trade: Podosomes as multipurpose organelles of monocytic cells. Cell. Mol. Life Sci..

[83] Alonso F., Spuul P., Génot E. (2020). Podosomes in endothelial cell--microenvironment interactions. Curr. Opin. Hematol..

[84] Jacob A., Prekeris R. (2015). The regulation of MMP targeting to invadopodia during cancer metastasis. Front. Cell Dev. Biol..

[85] Iizuka S., Abdullah C., Buschman M.D., Diaz B., Courtneidge S.A. (2016). The role of Tks adaptor proteins in invadopodia formation, growth and metastasis of melanoma. Oncotarget.

[86] Seals D.F., Azucena E.F., Pass I., Tesfay L., Gordon R., Woodrow M., Resau J.H., Courtneidge S.A. (2005). The adaptor protein Tks5/Fish is required for podosome formation and function, and for the protease-driven invasion of cancer cells. Cancer Cell.

[87] Destaing O., Planus E., Bouvard D., Oddou C., Badowski C., Bossy V., Raducanu A., Fourcade B., Albiges-Rizo C., Block M.R. (2010). β1A Integrin Is a Master Regulator of Invadosome Organization and Function. Mol. Biol. Cell.

[88] Daubon T., Spuul P., Alonso F., Fremaux I., Génot E. (2016). VEGF-A stimulates podosome-mediated collagen-IV proteolysis in microvascular endothelial cells. J. Cell Sci..

[89] Quintavalle M., Elia L., Condorelli G., Courtneidge S.A. (2010). MicroRNA control of podosome formation in vascular smooth muscle cells in vivo and in vitro. J. Cell Biol..

[90] Varon C., Tatin F., Moreau V., Obberghen-Schilling E.V., Fernandez-Sauze S., Reuzeau E., Kramer I., Génot E. (2006). Transforming Growth Factor β Induces Rosettes of Podosomes in Primary Aortic Endothelial Cells. Mol. Cell. Biol..

[91] Sa G., Liu Z., Ren J., Wan Q., Xiong X., Yu Z., Chen H., Zhao Y., He S. (2019). Keratinocyte growth factor (KGF) induces podosome formation via integrin-Erk1/2 signaling in human immortalized oral epithelial cells. Cell. Signal..

[92] Yamaguchi H., Pixley F., Condeelis J. (2006). Invadopodia and podosomes in tumor invasion. Eur. J. Cell Biol..

[93] Yamaguchi H., Lorenz M., Kempiak S., Sarmiento C., Coniglio S., Symons M., Segall J., Eddy R., Miki H., Takenawa T. (2005). Molecular mechanisms of invadopodium formation the role of the N-WASP–Arp2/3 complex pathway and cofilin. J. Cell Biol..

[94] Boateng L.R., Huttenlocher A. (2012). Spatiotemporal regulation of Src and its substrates at invadosomes. Eur. J. Cell Biol..

[95] Pan Y.-R., Chen C.-L., Chen H.-C. (2011). FAK is required for the assembly of podosome rosettes. J. Cell Biol..

[96] García E., Jones G.E., Machesky L.M., Antón I.M. (2012). WIP: WASP-interacting proteins at invadopodia and podosomes. Eur. J. Cell Biol..

[97] Bompard G., Caron E. (2004). Regulation of WASP/WAVE proteins making a long story short. J. Cell Biol..

[98] Murphy D.A., Courtneidge S.A. (2011). The “ins” and “outs” of podosomes and invadopodia: Characteristics, formation and function. Nat. Rev. Mol. Cell Biol..

[99] Schachtner H., Calaminus S.D.J., Thomas S.G., Machesky L.M. (2013). Podosomes in adhesion, migration, mechanosensing and matrix remodeling. Cytoskeleton.

[100] Linder S. (2009). Invadosomes at a glance. J. Cell Sci..

[101] Oser M., Dovas A., Cox D., Condeelis J. (2011). Nck1 and Grb2 localization patterns can distinguish invadopodia from podosomes. Eur. J. Cell Biol..

[102] Augoff K., Hryniewicz-Jankowska A., Tabola R. (2020). Invadopodia: Clearing the way for cancer cell invasion. Ann. Transl. Med..

[103] Walkiewicz K., Nowakowska-Zajdel E., Kozieł P., Muc-Wierzgoń M. (2018). The role of some ADAM-proteins and activation of the insulin growth factor-related pathway in colorectal cancer. Central Eur. J. Immunol..

[104] Sinderen M.V., Oyanedel J., Menkhorst E., Cuman C., Rainczuk K., Winship A., Salamonsen L., Edgell T., Dimitriadis E. (2017). Soluble Delta-like ligand 1 alters human endometrial epithelial cell adhesive capacity. Reprod. Fertil. Dev..

[105] Seike S., Takehara M., Kobayashi K., Nagahama M. (2019). Clostridium perfringens Delta-Toxin Damages the Mouse Small Intestine. Toxins.

[106] Dong W., Liu L., Dou Y., Xu M., Liu T., Wang S., Zhang Y., Deng B., Wang B., Cao H. (2018). Deoxycholic acid activates epidermal growth factor receptor and promotes intestinal carcinogenesis by ADAM17-dependent ligand release. J. Cell. Mol. Med..

[107] Miller M.A., Moss M.L., Powell G., Petrovich R., Edwards L., Meyer A.S., Griffith L.G., Lauffenburger D.A. (2015). Targeting autocrine HB-EGF signaling with specific ADAM12 inhibition using recombinant ADAM12 prodomain. Sci. Rep..

[108] Giebeler N., Zigrino P. (2016). A Disintegrin and Metalloprotease (ADAM): Historical Overview of Their Functions. Toxins.

[109] Feng Y., Tsai Y.-H., Xiao W., Ralls M.W., Stoeck A., Wilson C.L., Raines E.W., Teitelbaum D.H., Dempsey P.J. (2015). Loss of ADAM17-Mediated Tumor Necrosis Factor Alpha Signaling in Intestinal Cells Attenuates Mucosal Atrophy in a Mouse Model of Parenteral Nutrition. Mol. Cell. Biol..

[110] Hoffmann C., Mao X., Brown-Clay J., Moreau F., Al Absi A., Wurzer H., Sousa B., Schmitt F., Berchem G., Janji B. (2018). Hypoxia promotes breast cancer cell invasion through HIF-1α-mediated up-regulation of the invadopodial actin bundling protein CSRP2. Sci. Rep..

[111] Zacharias M., Brcic L., Eidenhammer S., Popper H. (2018). Bulk tumour cell migration in lung carcinomas might be more common than epithelial-mesenchymal transition and be differently regulated. BMC Cancer.

[112] Baik M., French B., Chen Y.-C., Byers J.T., Chen K.T., French S.W., Díaz B. (2019). Identification of invadopodia by TKS5 staining in human cancer lines and patient tumor samples. MethodsX.

[113] Ren X.L., Qiao Y.D., Li J.Y., Li X.M., Zhang D., Zhang X.J., Zhu X.H., Zhou W.J., Shi J., Wang W. (2018). Cortactin recruits FMNL2 to promote actin polymerization and endosome motility in invadopodia formation. Cancer Lett..

[114] Kedziora K.M., Leyton-Puig D., Argenzio E., Boumeester A.J., van Butselaar B., Yin T., Wu Y.I., van Leeuwen F.N., Innocenti M., Jalink K. (2016). Rapid Remodeling of Invadosomes by Gi-coupled Receptors: Dissecting the Role of Rho GTPases. J. Biol. Chem..

[115] Weidmann M.D., Surve C.R., Eddy R.J., Chen X., Gertler F.B., Sharma V.P., Condeelis J.S. (2016). MenaINV dysregulates cortactin phosphorylation to promote invadopodium maturation. Sci. Rep..

[116] Chen Y.-C., Baik M., Byers J.T., Chen K.T., French S.W., Díaz B. (2019). TKS5-positive invadopodia-like structures in human tumor surgical specimens. Exp. Mol. Pathol..

[117] Al Haddad M., El-Rif R., Hanna S., Jaafar L., Dennaoui R., Abdellatef S., Miskolci V., Cox D., Hodgson L., El-Sibai M. (2020). Differential regulation of rho GTPases during lung adenocarcinoma migration and invasion reveals a novel role of the tumor suppressor StarD13 in invadopodia regulation. Cell Commun. Signal..

[118] Ngan E., Stoletov K., Smith H.W., Common J., Muller W.J., Lewis J.D., Siegel P.M. (2017). LPP is a Src substrate required for invadopodia formation and efficient breast cancer lung metastasis. Nat. Commun..

[119] Mao L., Whitehead C.A., Paradiso L., Kaye A.H., Morokoff A.P., Luwor R.B., Stylli S.S. (2017). Enhancement of invadopodia activity in glioma cells by sublethal doses of irradiation and temozolomide. J. Neurosurg..

[120] Blouw B., Patel M., Iizuka S., Abdullah C., You W.K., Huang X., Li J.-L., Diaz B., Stallcup W.B., Courtneidge S.A. (2015). The Invadopodia Scaffold Protein Tks5 Is Required for the Growth of Human Breast Cancer Cells In Vitro and In Vivo. PLoS ONE.

[121] Bayarmagnai B., Perrin L., Pourfarhangi K.E., Graña X., Tüzel E., Gligorijevic B. (2019). Invadopodia-mediated ECM degradation is enhanced in the G1 phase of the cell cycle. J. Cell Sci..

[122] Li C.M.-C., Chen G., Dayton T.L., Kim-Kiselak C., Hoersch S., Whittaker C.A., Bronson R.T., Beer D.G., Winslow M.M., Jacks T. (2013). Differential Tks5 isoform expression contributes to metastatic invasion of lung adenocarcinoma. Genes Dev..

[123] Ribeiro A.L.R., da Costa N.M.M., de Siqueira A.S., Brasil da Silva W., da Silva Kataoka M.S., Jaeger R.G., de Melo Alves-Junior S., Smith A.M., de Jesus Viana Pinheiro J. (2016). Keratocystic odontogenic tumor overexpresses invadopodia-related proteins, suggesting invadopodia formation. Oral Surg. Oral Med. Oral Pathol. Oral Radiol..

[124] Shen Y., Wen Z., Li Y., Matteson E.L., Hong J., Goronzy J.J., Weyand C.M. (2017). Metabolic control of the scaffold protein TKS5 in tissue-invasive, proinflammatory T cells. Nat. Immunol..

[125] Cejudo-Martin P., Yuen A., Vlahovich N., Lock P., Courtneidge S.A., Díaz B. (2014). Genetic Disruption of the Sh3pxd2a Gene Reveals an Essential Role in Mouse Development and the Existence of a Novel Isoform of Tks5. PLoS ONE.

[126] Peláez R., Morales X., Salvo E., Garasa S., de Solórzano C.O., Martínez A., Larrayoz I.M., Rouzaut A. (2017). β3 integrin expression is required for invadopodia-mediated ECM degradation in lung carcinoma cells. PLoS ONE.

[127] Courtneidge S.A. (2012). Cell migration and invasion in human disease: The Tks adaptor proteins. Biochem. Soc. Trans..

[128] Gonzalez-Avila G., Sommer B., Mendoza-Posada D.A., Ramos C., Garcia-Hernandez A.A., Falfan-Valencia R. (2019). Matrix metalloproteinases participation in the metastatic process and their diagnostic and therapeutic applications in cancer. Crit. Rev. Oncol. Hematol..

[129] Hoshino D., Kirkbride K.C., Costello K., Clark E.S., Sinha S., Grega-Larson N., Tyska M.J., Weaver A.M. (2013). Exosome Secretion Is Enhanced by Invadopodia and Drives Invasive Behavior. Cell Rep..

[130] Pant S., Hilton H., Burczynski M.E. (2012). The multifaceted exosome: Biogenesis, role in normal and aberrant cellular function, and frontiers for pharmacological and biomarker opportunities. Biochem. Pharmacol..

[131] Yang H., Villani R.M., Wang H., Simpson M.J., Roberts M.S., Tang M., Liang X. (2018). The role of cellular reactive oxygen species in cancer chemotherapy. J. Exp. Clin. Cancer Res. CR.

[132] Chen J., Wang Y., Zhang W., Zhao D., Zhang L., Fan J., Li J., Zhan Q. (2020). Membranous NOX5-derived ROS oxidizes and activates local Src to promote malignancy of tumor cells. Signal Transduct. Target. Ther..

[133] Block K., Gorin Y. (2012). Aiding and abetting roles of NOX oxidases in cellular transformation. Nat. Rev. Cancer.

[134] Blaser H., Dostert C., Mak T.W., Brenner D. (2016). TNF and ROS Crosstalk in Inflammation. Trends Cell Biol..

[135] Singh A., Kukreti R., Saso L., Kukreti S. (2019). Oxidative Stress: A Key Modulator in Neurodegenerative Diseases. Molecules.

[136] Coant N., Mkaddem S.B., Pedruzzi E., Guichard C., Tréton X., Ducroc R., Freund J.-N., Cazals-Hatem D., Bouhnik Y., Woerther P.-L. (2010). NADPH Oxidase 1 Modulates WNT and NOTCH1 Signaling To Control the Fate of Proliferative Progenitor Cells in the Colon. Mol. Cell. Biol..

[137] Smallwood M.J., Nissim A., Knight A.R., Whiteman M., Haigh R., Winyard P.G. (2018). Oxidative stress in autoimmune rheumatic diseases. Free Radic. Biol. Med..

[138] Kallenborn-Gerhardt W., Schröder K., Geisslinger G., Schmidtko A. (2013). NOXious signaling in pain processing. Pharmacol. Ther..

[139] Koju N., Taleb A., Zhou J., Lv G., Yang J., Cao X., Lei H., Ding Q. (2019). Pharmacological strategies to lower crosstalk between nicotinamide adenine dinucleotide phosphate (NADPH) oxidase and mitochondria. Biomed. Pharmacother..

[140] Leto T.L., Morand S., Hurt D., Ueyama T. (2009). Targeting and Regulation of Reactive Oxygen Species Generation by Nox Family NADPH Oxidases. Antioxid. Redox Signal..

[141] Altenhöfer S., Kleikers P.W.M., Radermacher K.A., Scheurer P., Rob Hermans J.J., Schiffers P., Ho H., Wingler K., Schmidt H.H.H.W. (2012). The NOX toolbox: Validating the role of NADPH oxidases in physiology and disease. Cell. Mol. Life Sci..

[142] Lambeth J.D., Kawahara T., Diebold B. (2007). Regulation of Nox and Duox enzymatic activity and expression. Free Radic. Biol. Med..

[143] Petry A., Weitnauer M., Görlach A. (2009). Receptor Activation of NADPH Oxidases. Antioxid. Redox Signal..

[144] Mortezaee K. (2018). Nicotinamide adenine dinucleotide phosphate (NADPH) oxidase (NOX) and liver fibrosis: A review. Cell Biochem. Funct..

[145] Drummond G.R., Selemidis S., Griendling K.K., Sobey C.G. (2011). Combating oxidative stress in vascular disease: NADPH oxidases as therapeutic targets. Nat. Rev. Drug Discov..

[146] Pani G., Galeotti T., Chiarugi P. (2010). Metastasis: Cancer cell’s escape from oxidative stress. Cancer Metastasis Rev..

[147] Caires-Dos-Santos L., da Silva S.V., Smuczek B., de Siqueira A.S., Cruz K.S.P., Barbuto J.A.M., Augusto T.M., Freitas V.M., Carvalho H.F., Jaeger R.G. (2020). Laminin-derived peptide C16 regulates Tks expression and reactive oxygen species generation in human prostate cancer cells. J. Cell. Physiol..

[148] Petushkova A.I., Zamyatnin A.A. (2020). Redox-Mediated Post-Translational Modifications of Proteolytic Enzymes and Their Role in Protease Functioning. Biomolecules.

[149] Finkel T. (2011). Signal transduction by reactive oxygen species. J. Cell Biol..

[150] Miki H., Funato Y. (2012). Regulation of intracellular signalling through cysteine oxidation by reactive oxygen species. J. Biochem..

[151] Hurd T.R., DeGennaro M., Lehmann R. (2012). Redox regulation of cell migration and adhesion. Trends Cell Biol..

[152] Milkovic L., Cipak Gasparovic A., Cindric M., Mouthuy P.-A., Zarkovic N. (2019). Short Overview of ROS as Cell Function Regulators and Their Implications in Therapy Concepts. Cells.

[153] Szeder B., Tárnoki-Zách J., Lakatos D., Vas V., Kudlik G., Merő B., Koprivanacz K., Bányai L., Hámori L., Róna G. (2019). Absence of the Tks4 Scaffold Protein Induces Epithelial-Mesenchymal Transition-Like Changes in Human Colon Cancer Cells. Cells.

[154] Dongre A., Weinberg R.A. (2019). New insights into the mechanisms of epithelial–mesenchymal transition and implications for cancer. Nat. Rev. Mol. Cell Biol..

[155] Horejs C.-M. (2016). Basement membrane fragments in the context of the epithelial-to-mesenchymal transition. Eur. J. Cell Biol..

[156] Song J., Wang W., Wang Y., Qin Y., Wang Y., Zhou J., Wang X., Zhang Y., Wang Q. (2019). Epithelial-mesenchymal transition markers screened in a cell-based model and validated in lung adenocarcinoma. BMC Cancer.

[157] Clapéron A., Mergey M., Nguyen Ho-Bouldoires T.H., Vignjevic D., Wendum D., Chrétien Y., Merabtene F., Frazao A., Paradis V., Housset C. (2014). EGF/EGFR axis contributes to the progression of cholangiocarcinoma through the induction of an epithelial-mesenchymal transition. J. Hepatol..

[158] Cheng J.-C., Auersperg N., Leung P.C.K. (2012). EGF-Induced EMT and Invasiveness in Serous Borderline Ovarian Tumor Cells: A Possible Step in the Transition to Low-Grade Serous Carcinoma Cells?. PLoS ONE.

[159] Jiao L., Li D.-D., Yang C.-L., Peng R.-Q., Guo Y.-Q., Zhang X.-S., Zhu X.-F. (2016). Reactive oxygen species mediate oxaliplatin-induced epithelial-mesenchymal transition and invasive potential in colon cancer. Tumor Biol..

[160] Jung S.-H., Kim S.-M., Lee C.-E. (2016). Mechanism of suppressors of cytokine signaling 1 inhibition of epithelial-mesenchymal transition signaling through ROS regulation in colon cancer cells: Suppression of Src leading to thioredoxin up-regulation. Oncotarget.

[161] Nakaya Y., Sheng G. (2013). EMT in developmental morphogenesis. Cancer Lett..

[162] Kim D.H., Xing T., Yang Z., Dudek R., Lu Q., Chen Y.-H. (2018). Epithelial Mesenchymal Transition in Embryonic Development, Tissue Repair and Cancer: A Comprehensive Overview. J. Clin. Med..

[163] Maas S.M., Kayserili H., Lam J., Apak M.Y., Hennekam R.C.M. (2004). Further delineation of Frank–ter Haar syndrome. Am. J. Med. Genet. Part A.

[164] Iqbal Z., Cejudo-Martin P., de Brouwer A., van der Zwaag B., Ruiz-Lozano P., Scimia M.C., Lindsey J.D., Weinreb R., Albrecht B., Megarbane A. (2010). Disruption of the Podosome Adaptor Protein TKS4 (SH3PXD2B) Causes the Skeletal Dysplasia, Eye, and Cardiac Abnormalities of Frank-Ter Haar Syndrome. Am. J. Hum. Genet..

[165] Bendon C.L., Fenwick A.L., Hurst J.A., Nürnberg G., Nürnberg P., Wall S.A., Wilkie A.O., Johnson D. (2012). Frank-ter Haar syndrome associated with sagittal craniosynostosis and raised intracranial pressure. BMC Med. Genet..

[166] Dülk M., Kudlik G., Fekete A., Ernszt D., Kvell K., Pongrácz J.E., Merő B.L., Szeder B., Radnai L., Geiszt M. (2016). The scaffold protein Tks4 is required for the differentiation of mesenchymal stromal cells (MSCs) into adipogenic and osteogenic lineages. Sci. Rep..

[167] Dushnik-Levinson M., Benvenisty N. (1995). Embryogenesis in vitro: Study of Differentiation of Embryonic Stem Cells. Neonatology.

[168] Kurosaka S., Kashina A. (2008). Cell biology of embryonic migration. Birth Defects Res. Part C Embryo Today Rev..

[169] Pellettieri J., Alvarado A.S. (2007). Cell Turnover and Adult Tissue Homeostasis: From Humans to Planarians. Annu. Rev. Genet..

[170] Post Y., Clevers H. (2019). Defining Adult Stem Cell Function at Its Simplest: The Ability to Replace Lost Cells through Mitosis. Cell Stem Cell.

[171] Chen Q., Shou P., Zheng C., Jiang M., Cao G., Yang Q., Cao J., Xie N., Velletri T., Zhang X. (2016). Fate decision of mesenchymal stem cells: Adipocytes or osteoblasts?. Cell Death Differ..

[172] Medina-Gomez G., Gray S.L., Yetukuri L., Shimomura K., Virtue S., Campbell M., Curtis R.K., Jimenez-Linan M., Blount M., Yeo G.S.H. (2007). PPAR gamma 2 Prevents Lipotoxicity by Controlling Adipose Tissue Expandability and Peripheral Lipid Metabolism. PLoS Genet..

[173] Vas V., Háhner T., Kudlik G., Ernszt D., Kvell K., Kuti D., Kovács K.J., Tóvári J., Trexler M., Merő B.L. (2019). Analysis of Tks4 Knockout Mice Suggests a Role for Tks4 in Adipose Tissue Homeostasis in the Context of Beigeing. Cells.

[174] Murphy D.A., Diaz B., Bromann P.A., Tsai J.H., Kawakami Y., Maurer J., Stewart R.A., Izpisúa-Belmonte J.C., Courtneidge S.A. (2011). A Src-Tks5 Pathway Is Required for Neural Crest Cell Migration during Embryonic Development. PLoS ONE.

[175] Liu Y.-J., Liu X.-G., Wang L., Dina C., Yan H., Liu J.-F., Levy S., Papasian C.J., Drees B.M., Hamilton J.J. (2008). Genome-wide association scans identified CTNNBL1 as a novel gene for obesity. Hum. Mol. Genet..

[176] Vogel C.I., Greene B., Scherag A., Müller T.D., Friedel S., Grallert H., Heid I.M., Illig T., Wichmann H.-E., Schäfer H. (2009). Non-replication of an association of CTNNBL1polymorphisms and obesity in a population of Central European ancestry. BMC Med. Genet..

[177] Cleal L., Aldea T., Chau Y.-Y. (2017). Fifty shades of white: Understanding heterogeneity in white adipose stem cells. Adipocyte.

[178] Kajimura S., Spiegelman B.M., Seale P. (2015). Brown and Beige Fat: Physiological Roles beyond Heat Generation. Cell Metab..

[179] Vas V., Kovács T., Körmendi S., Bródy A., Kudlik G., Szeder B., Mező D., Kállai D., Koprivanacz K., Merő B.L. (2019). Significance of the Tks4 scaffold protein in bone tissue homeostasis. Sci. Rep..

[180] Langdahl B., Ferrari S., Dempster D.W. (2016). Bone modeling and remodeling: Potential as therapeutic targets for the treatment of osteoporosis. Ther. Adv. Musculoskelet. Dis..

[181] Ter Haar B., Hamel B., Hendriks J., de Jager J., Opitz J.M. (1982). Melnick-Needles syndrome: Indication for an autosomal recessive form. Am. J. Med. Genet..

[182] Frank Y., Ziprkowski M., Romano A., Stein R., Katznelson M.B., Cohen B., Goodman R.M. (1973). Megalocornea associated with multiple skeletal anomalies: A new genetic syndrome?. J. Genet. Hum..

[183] Zrhidri A., Jaouad I.C., Lyahyai J., Raymond L., Egéa G., Taoudi M., El Mouatassim S., Sefiani A. (2017). Identification of two novel SH3PXD2B gene mutations in Frank-Ter Haar syndrome by exome sequencing: Case report and review of the literature. Gene.

[184] Ratukondla B., Prakash S., Reddy S., Puthuran G.V., Kannan N.B., Pillai M.R. (2020). A Rare Case Report of Frank Ter Haar Syndrome in a Sibling Pair Presenting With Congenital Glaucoma. J. Glaucoma.

[185] Durand B., Stoetzel C., Schaefer E., Calmels N., Scheidecker S., Kempf N., De Melo C., Guilbert A.-S., Timbolschi D., Donato L. (2020). A severe case of Frank-ter Haar syndrome and literature review: Further delineation of the phenotypical spectrum. Eur. J. Med. Genet..

[186] Wilson G.R., Sunley J., Smith K.R., Pope K., Bromhead C.J., Fitzpatrick E., Di Rocco M., van Steensel M., Coman D.J., Leventer R.J. (2014). Mutations in SH3PXD2B cause Borrone dermato-cardio-skeletal syndrome. Eur. J. Hum. Genet..

[187] Kanai F., Liu H., Field S.J., Akbary H., Matsuo T., Brown G.E., Cantley L.C., Yaffe M.B. (2001). The PX domains of p47phox and p40phox bind to lipid products of PI(3)K. Nat. Cell Biol..

[188] Chang T.C., Bauer M., Puerta H.S., Greenberg M.B., Cavuoto K.M. (2017). Ophthalmic findings in Frank-ter Haar syndrome: Report of a sibling pair. J. Am. Assoc. Pediatr. Ophthalmol. Strabismus.

[189] Mao M., Solivan-Timpe F., Roos B.R., Mullins R.F., Oetting T.A., Kwon Y.H., Brzeskiewicz P.M., Stone E.M., Alward W.L.M., Anderson M.G. (2012). Localization of SH3PXD2B in human eyes and detection of rare variants in patients with anterior segment diseases and glaucoma. Mol. Vis..

[190] Bernstein H.-G., Keilhoff G., Dobrowolny H., Lendeckel U., Steiner J. (2020). From putative brain tumor marker to high cognitive abilities: Emerging roles of a disintegrin and metalloprotease (ADAM) 12 in the brain. J. Chem. Neuroanat..

[191] Malinin N.L., Wright S., Seubert P., Schenk D., Griswold-Prenner I. (2005). Amyloid-β neurotoxicity is mediated by FISH adapter protein and ADAM12 metalloprotease activity. Proc. Natl. Acad. Sci. USA.

[192] Xiang Y., Cheng Y., Li X., Li Q., Xu J., Zhang J., Liu Y., Xing Q., Wang L., He L. (2013). Up-Regulated Expression and Aberrant DNA Methylation of LEP and SH3PXD2A in Pre-Eclampsia. PLoS ONE.

[193] Patel A., Dash P.R. (2012). Formation of atypical podosomes in extravillous trophoblasts regulates extracellular matrix degradation. Eur. J. Cell Biol..

[194] Mehes E., Barath M., Gulyas M., Bugyik E., Geiszt M., Szoor A., Lanyi A., Czirok A. (2019). Enhanced endothelial motility and multicellular sprouting is mediated by the scaffold protein TKS4. Sci. Rep..

